# Trajectories of cortical structures associated with stress across adolescence: a bivariate latent change score approach

**DOI:** 10.1111/jcpp.13793

**Published:** 2023-03-29

**Authors:** Tochukwu Nweze, Tobias Banaschewski, Cyracius Ajaelu, Chukwuemeka Okoye, Michael Ezenwa, Robert Whelan, Dimitri Papadopoulos Orfanos, Arun L.W. Bokde, Sylvane Desrivières, Antoine Grigis, Hugh Garavan, Penny Gowland, Andreas Heinz, Rüdiger Brühl, Jean‐Luc Martinot, Marie‐Laure Paillère Martinot, Eric Artiges, Frauke Nees, Tomáš Paus, Luise Poustka, Sarah Hohmann, Sabina Millenet, Juliane H. Fröhner, Michael N. Smolka, Henrik Walter, Gunter Schumann, Jamie L. Hanson, IMAGEN Consortium

**Affiliations:** ^1^ MRC Cognition and Brain Sciences Unit University of Cambridge Cambridge UK; ^2^ Department of Psychology University of Nigeria Nsukka Nigeria; ^3^ Department of Child and Adolescent Psychiatry and Psychotherapy, Central Institute of Mental Health, Medical Faculty Mannheim Heidelberg University Mannheim Germany; ^4^ Department of Psychology Nnamdi Azikiwe University Awka Nigeria; ^5^ School of Psychology Global Brain Health Institute, Trinity College Dublin Dublin Ireland; ^6^ NeuroSpin, CEA Université Paris‐Saclay Gif‐sur‐Yvette France; ^7^ Discipline of Psychiatry School of Medicine, Trinity College Institute of Neuroscience, Trinity College Dublin Dublin Ireland; ^8^ SGDP Centre Institute of Psychiatry, Psychology & Neuroscience, SGDP Centre, King's College London London UK; ^9^ Departments of Psychiatry and Psychology University of Vermont Burlington VT USA; ^10^ Sir Peter Mansfield Imaging Centre School of Physics and Astronomy, University of Nottingham, University Park Nottingham UK; ^11^ Department of Psychiatry and Psychotherapy CCM Charité – Universitätsmedizin Berlin, Corporate Member of Freie Universität Berlin, Humboldt‐Universität zu Berlin Berlin Germany; ^12^ Berlin Institute of Health Berlin Germany; ^13^ Physikalisch‐Technische Bundesanstalt (PTB) Braunschweig and Berlin Germany; ^14^ CNRS, Institut National de la Santé et de la Recherche Médicale Université Paris‐Saclay Gif‐sur‐Yvette France; ^15^ Centre Borelli, INSERM U1299 ‘Trajectoires Développementales et Psychiatrie’ Ecole Normale Supérieure Paris‐Saclay Gif‐sur‐Yvette France; ^16^ CNRS, Institut National de la Santé et de la Recherche Médicale Université Paris‐Saclay Paris France; ^17^ Centre Borelli, INSERM U1299 ‘Trajectoires Développementales et Psychiatrie’ Ecole Normale Supérieure Paris‐Saclay Paris France; ^18^ Department of Child and Adolescent Psychiatry, Pitié‐Salpétriere Hospital, AP‐HP Sorbonne Université Paris France; ^19^ CNRS, Institut National de la Santé et de la Recherche Médicale Université Paris‐Saclay Etampes France; ^20^ Ecole Normale Supérieure Paris‐Saclay, Centre Borelli, INSERM U1299 ‘Trajectoires Développementales et Psychiatrie’ Etampes France; ^21^ CH Bartélémy Durand Etampes France; ^22^ Institute of Cognitive and Clinical Neuroscience, Central Institute of Mental Health, Medical Faculty Mannheim Heidelberg University Mannheim Germany; ^23^ Institute of Medical Psychology and Medical Sociology University Medical Center Schleswig Holstein, Kiel University Kiel Germany; ^24^ Department of Psychiatry, Faculty of Medicine, Centre Hospitalier Universitaire Sainte‐Justine University of Montreal Montreal QC Canada; ^25^ Departments of Psychiatry and Psychology University of Toronto Toronto ON Canada; ^26^ Department of Child and Adolescent Psychiatry and Psychotherapy University Medical Centre Göttingen Göttingen Germany; ^27^ Department of Psychiatry and Neuroimaging Center Technische Universität Dresden Dresden Germany; ^28^ Centre for Population Neuroscience and Stratified Medicine (PONS), Department of Psychiatry and Neuroscience Charité Universitätsmedizin Berlin Berlin Germany; ^29^ Centre for Population Neuroscience and Precision Medicine (PONS), Institute for Science and Technology of Brain‐inspired Intelligence (ISTBI) Fudan University Shanghai China; ^30^ Department of Psychology University of Pittsburgh Pittsburgh PA USA; ^31^ Learning Research & Development Center University of Pittsburgh Pittsburgh PA USA

**Keywords:** Stress, cortical development, cognitive functioning, longitudinal models, bivariate latent change score model, longitudinal mediation analysis

## Abstract

**Background:**

Stress exposure in childhood and adolescence has been linked to reductions in cortical structures and cognitive functioning. However, to date, most of these studies have been cross‐sectional, limiting the ability to make long‐term inferences, given that most cortical structures continue to develop through adolescence.

**Methods:**

Here, we used a subset of the IMAGEN population cohort sample (*N* = 502; assessment ages: 14, 19, and 22 years; mean age: 21.945 years; *SD* = 0.610) to understand longitudinally the long‐term interrelations between stress, cortical development, and cognitive functioning. To these ends, we first used a latent change score model to examine four bivariate relations – assessing individual differences in change in the relations between adolescent stress exposure and volume, surface area, and cortical thickness of cortical structures, as well as cognitive outcomes. Second, we probed for indirect neurocognitive effects linking stress to cortical brain structures and cognitive functions using rich longitudinal mediation modeling.

**Results:**

Latent change score modeling showed that greater baseline adolescence stress at age 14 predicted a small reduction in the right anterior cingulate volume (Std. *β* = −.327, *p* = .042, 95% CI [−0.643, −0.012]) and right anterior cingulate surface area (Std. *β =* −.274, *p* = .038, 95% CI [−0.533, −0.015]) across ages 14–22. These effects were very modest in nature and became nonsignificant after correcting for multiple comparisons. Our longitudinal analyses found no evidence of indirect effects in the two neurocognitive pathways linking adolescent stress to brain and cognitive outcomes.

**Conclusion:**

Findings shed light on the impact of stress on brain reductions, particularly in the prefrontal cortex that have consistently been implicated in the previous cross‐sectional studies. However, the magnitude of effects observed in our study is smaller than that has been reported in past cross‐sectional work. This suggests that the potential impact of stress during adolescence on brain structures may likely be more modest than previously noted.

## Introduction

Stress in childhood and adolescence has been associated with poorer developmental outcomes. Numerous studies have linked stress during development to poorer mental health (Gilbert et al., 2009), problem behaviors (Golm et al., 2020; Sonuga‐Barke et al., 2017), cognitive deficits (Guinosso, Johnson, & Riley, 2015), and structural reductions in the brain (McLaughlin, Weissman, & Bitrán, 2019; Teicher, Samson, Anderson, & Ohashi, 2016). Although these findings are robust, very few studies have longitudinally modeled the individual differences in change in these relations between stress exposure, brain development, and cognitive functioning (i.e., how stress exposure at one timepoint predicts change in brain development or cognitive functioning between two timepoints). Given that all those exposed to stress in childhood or adolescence do not go on to develop challenges (Ellis et al., 2020), it is imperative to understand the different developmental impacts of stress across the life span. Thus, using different longitudinal models, the primary aim of this empirical study is to extend the results found in past cross‐sectional projects and more intricately understand long‐term interrelations between stress exposure, brain development, and cognitive functioning. Here, we defined stress as persistent adverse life events that deviate from the expected environment, but which have a lesser degree of severity than adversities measured in previous investigations (e.g., institutionalization, extreme neglect, and child abuse).

The effects of stress on cortical and subcortical structures are well documented (Hanson et al., 2012, 2015; Hodel et al., 2015; Luby, Tillman, & Barch, 2019; Lupien et al., 2011; Sheridan, Fox, Zeanah, McLaughlin, & Nelson, 2012; Tottenham et al., 2010). For example, environmental deprivation, e.g., institutionalization or poverty (Sheridan & McLaughlin, 2014), as well as mild and uncontrollable stress (Arnsten, 2009; Lupien, McEwen, Gunnar, & Heim, 2009), have been linked to reductions in frontoparietal brain regions, including facets of the prefrontal regions. The effects of chronic stress on the frontoparietal regions are present at adolescence (Arnsten, 2009; Lupien et al., 2009), with child abuse (Gold et al., 2016; Hanson et al., 2010; Kelly et al., 2013; Thomaes et al., 2010) or child institutionalization (Hodel et al., 2015; Mackes et al., 2020; McLaughlin et al., 2014) being related to structural brain reductions in various prefrontal and parietal regions. Other studies that examined the association between cortical structures and mild childhood stressors using the early‐life stress questionnaire found that stress is linked to smaller anterior cingulate cortex (Baker et al., 2012; Cohen et al., 2006). Given that these cortical structures, especially prefrontal cortex subregions, continue to develop during adolescence (Tamnes et al., 2017), it may make these regions more susceptible to brain reductions induced by stress compared with other brain areas.

Similar to relations with cortical development, previous studies have shown that chronic stress across childhood and adolescence exacts negative effects on cognitive functioning (Nweze, Ezenwa, Ajaelu, & Okoye, 2023). There may be common mechanisms through which the effects of stress manifest on both cognitive and neurodevelopmental processes (Arnsten, 2009). Studies have consistently linked child institutionalization to poorer performance on cognitive tasks (Golm et al., 2020; Merz, Harlé, Noble, & McCall, 2016), including executive functions, working memory, and decision‐making across the life span. Importantly, different cortical structures (Oschwald et al., 2020), particularly, the prefrontal regions (Friedman & Robbins, 2022; Stuss, 2011), are similarly implicated in these higher order cognitive processes ranging from planning, decision‐making, attention, memory, and executive functions. This suggests a potential pathway through which the effects of stress may impact cognition and brain development. Previous studies have suggested that exposure to overwhelming stress may trigger accelerated loss of prefrontal cognitive abilities, while chronic stress has been linked to structural reductions in prefrontal dendrites (Arnsten, 2009). Functional imaging techniques provide unique methods of establishing this pathway of interrelation between stress, cognition, and brain development. For example, in work focused on reward processing and decision‐making (Birn, Roeber, & Pollak, 2017), individuals who experienced high levels of community stress (i.e., day‐to‐day stressors) showed reduced activation in the posterior cingulate, precuneus, and insula when processing cues about potential loss and rewards and greater activation in the inferior frontal regions when experiencing loss. This result suggests that childhood stress may compromise learning abilities, specifically being able to adjust behaviors after one's loss; rather than global deficits in how rewards and punishments are processed. Other studies examining network connectivity (Philip et al., 2013; Teicher, Anderson, Ohashi, & Polcari, 2014) in maltreated children have observed altered connectivity in many frontoparietal networks, including the cingulate, prefrontal cortex, precuneus, and insula. These brain areas are critical to higher order cognitive abilities, such as working memory, emotion regulation, performance monitoring, and self‐awareness (Cavanna & Trimble, 2006; Haber & Knutson, 2009; Lara & Wallis, 2015). Examined collectively, these findings suggest a potential common pathway through which stress exposure alters typical trajectories of brain and cognitive development.

While evidence linking chronic stress to cognitive deficits and brain reductions across human development is well established, important gaps that hinder the long‐term inferences of these associations and interrelations still exist. First, most studies examining the effects of chronic stress have been cross‐sectional and consequently limit the ability to make long‐term inferences. Some other studies (e.g., Golm et al., 2020; Mackes et al., 2020; Mehta et al., 2009) have attempted to use a pseudo‐experimental design by following a group of institutionalized children who were adopted to more enriched homes during very early childhood. Yet, such studies are often limited because they involve mostly small samples who had faced extreme adversities and often report high levels of psychopathology. In contrast, the use of population data samples offers an opportunity for researchers to establish stronger links on the effects of stress and adversity due to the availability of large, longitudinal datasets (Rosenberg, Casey, & Holmes, 2018). Yet very few published studies have utilized these longitudinal datasets to understand individual differences in change between chronic stress and brain development. That is, to what extent changes in brain development between two or more assessment timepoints are related to the effects of childhood or adolescence stress exposure. Second, few studies examining the neurobiological effects of stress and adversity have simultaneously examined the multiple morphometric measures of the brain (i.e., volume, surface area, and cortical thickness). The preponderance of work has focused on brain volume, surface area, or cortical thickness in isolation (cf., Gehred et al., 2021; Mackes et al., 2020; Rinne‐Albers et al., 2020). Structural and cellular differences between these brain morphometrics warrant that they may be considered or investigated separately. For example, brain volume is a product of surface area and cortical thickness (Panizzon et al., 2009), with the size of the surface area believed to be driven by the laminar structure of the cortex (i.e., number of columns), while cortical thickness is influenced by the number of cells within columns (Panizzon et al., 2009; Rakic, 1988). These morphometrics also have different growth trajectories (Raznahan et al., 2011), and past empirical studies have shown that reduction in the cortical brain volume is almost attributable to variations in the surface area rather than cortical thickness (Im et al., 2008; Storsve et al., 2014) suggesting their distinct features and the need to investigate the effects of stress and adversity on each specific morphometric. In relation to stress, one of the few studies that investigated these three brain morphometrics (i.e., volume, surface area, and cortical thickness) showed in an exploratory analysis that adversity exposure was associated with reductions in the different portions of the prefrontal cortex volumes; particularly notable, these reductions in brain volumes were largely driven by variability in the surface areas than in the cortical thickness (Hodel et al., 2015). A further exploration, using longitudinal models to examine and compare these brain morphometrics, may therefore provide additional insight into the effects of stress and adversity on the specific brain morphometrics. Lastly, although studies have attempted to establish inter‐correlations existing between stress, cognition, and brain development through several methods (e.g., functional imaging analysis), no study, to our knowledge, has modeled the longitudinal relations existing among these constructs.

The purpose of the current study is, therefore, threefold. First, to use longitudinal models (latent change scores) to extend the robust cross‐sectional studies linking chronic stress to reductions in brain and cognitive development. This change score model can quantify the individual differences in change observed over time among those exposed to chronic stress. This is pertinent given that previous studies have indicated potential nuanced and heterogeneous effects of stress on cortical structures (Mackes et al., 2020), neural connectivity (Ellwood‐Lowe, Whitfield‐Gabrieli, & Bunge, 2021), and cognitive outcomes (Fields et al., 2021; Nweze, Nwoke, Nwufo, Aniekwu, & Lange, 2020; Nweze, Ezenwa, Ajaelu, Hanson, & Okoye, 2022; Young et al., 2022). Thus, teasing out the portions of developmental trajectories when deficits, or compensatory benefits, emerge in the brain or cognitive functioning could be very crucial in understanding the effects of stress across adolescence. Latent change models also enable us to establish the bidirectionality (i.e., how stress affects brain and cognitive functions, versus how brain and cognitive outcomes affect stress on the other hand). This may provide additional insight into the complex effects of stress on brain and cognitive development. Most previous studies on stress were centered on the more intuitive framework of effects of stress on developmental processes (e.g., brain development and cognitive functioning). Thus, we know very little about how stress‐associated changes in these developmental processes might affect the subsequent perception of prolonged stress given the paucity of studies that have used bidirectional analytic strategies. The few studies that have investigated the role of brain on perceived stress have implicated reductions in the brain's frontal regions, particularly the prefrontal cortex (McEwen & Morrison, 2013; Michalski et al., 2017; Moreno, Bruss, & Denburg, 2017). Second, in examining the link between chronic stress and brain development, we capture all three morphometric measures of brain assessments (i.e., volume, surface area, and cortical thickness). A simultaneous investigation of all three outcomes will enable us to disentangle the effects of stress on relatively established brain volumes, compared to the more fine‐grained surface area and cortical thickness. In other words, we can probe if chronic stress has equal or disproportionate effects on brain volume, surface area, and cortical thickness. Lastly, we attempt to establish the interrelations between chronic stress, cortical brain structures, and two cognitive processes of spatial working memory and decision‐making. We were specifically interested in examining the mediating effects of both brain and cognitive outcomes using complete longitudinal mediation models. This model tests whether any mediating effects exist in our data for neurobiology or cognition, and if so, further disentangles specific mediating paths. For example, our models allow us to understand if stress predicts brain reductions which in turn predicts cognitive deficits, or in contrast, if stress predicts cognitive deficits which in turn predicts brain reductions. Testing these two neurocognitive pathways to stress would enable a more holistic appreciation of the mediating interrelations between stress, brain, and cognition given previous studies that have suggested common mechanisms through which the effects of stress manifests on both cognitive and neurodevelopmental processes (Arnsten, 2009). By testing the two pathways, we can identify the specific neurocognitive mechanism driving the mediation effects or rule out evidence of mediating effects in the two neurocognitive pathways. As noted previously (Jose, 2016), such an approach would be more persuasive and complete, compared to a mediation test of a single pathway. Given findings from previous cross‐sectional studies, we predict that stress will lead to decrease change in cortical structures across adolescence and these reductions will be equally distributed across the three brain morphometric outcomes we examined. For mediation models, we predict a mediating effect in the two neurocognitive pathways; in the first pathway, we hypothesize that stress at time 1 will predict changes in brain development at time 2 which will predict change in cognitive functioning (measured by the Cambridge gambling task and spatial working memory task) at time 3. Alternatively, we also hypothesize for the second mediation pathway, that stress at time 1 will predict change in cognitive abilities at time 2 and this will in turn predict change in brain development at time 3.

## Methods

### Participants

Data were obtained from the IMAGEN project – a European multicenter longitudinal cohort study that is primarily focused on understanding how biological, psychological, and environmental factors interact to shape the brain development and mental health of young people. (See Schumann et al., 2010 for detailed description of study protocols.) Data were first collected when the participants were aged 14, but subsequent follow‐up assessments have taken place at ages 16, 19, and 22.

Our analytic sample consists of 502 participants (Male = 283; Female = 219) who completed all MRI scans, as well as behavioral and cognitive assessments at ages 14 (mean = 13.978 years; *SD* = 0.518), 19 (mean = 18.483 years; *SD* = 0.725), and 22 (mean = 21.945 years; *SD* = 0.610). Data obtained at age 16 were not included in the analysis because there was no MRI assessment at this time point.

### Measures

#### Chronic stress

Participants completed the 39‐item life events questionnaire (Newcomb, Huba, & Bentler, 1981) and the revised Olweus bully/victim questionnaire (Olweus, 2006) at ages 14, 19, and 22. These measures have been used in previous studies that examined the effects of stress in this cohort sample (Galinowski et al., 2015; Gollier‐Briant et al., 2016; Mackey et al., 2017). The life events questionnaire has seven subscales: parents and family, accident and illness, sexuality, autonomy, deviance, relocation, and distress. In the first assessment (age 14), the participants were asked about their lifetime exposure to these events in the questionnaire. But in the follow‐up assessments at ages 19 and 22, the participants were asked about their exposure to the events since their last IMAGEN visit. In this study, only the negative life events captured in two subscales (parents/family and accident/illness) consisting of nine items were included in the analysis. These two subscales were used in this study because first, they broadly reflect on general adverse experiences involving the individuals or their immediate family. Second, they represent stress variables that have been operationalized within the adversity frameworks in the IMAGEN cohort. Lastly, previous studies using the IMAGEN dataset have used these subscales when defining stress or adversity exposure (Galinowski et al., 2015; Gollier‐Briant et al., 2016; Mackey et al., 2017). Relatedly, the bully/victim questionnaire is a widely used questionnaire for the assessment of bullying either as a victim or as a perpetrator in school‐aged children. For each of the assessment time, the participants were asked if they have been exposed to bullying in the last 6 months. We included only the victim subscale which has six‐item questions because they reflect on exposure to stress to an individual. See Appendix S1 for all the item questions in both the subscales of life events questionnaire and bullying questionnaire used in the study. Exposures to any of the item questions in the selected subscales in life events questionnaire or bully/victim questionnaire were initially coded 1 or 0 if no exposure was reported. We transformed all the individual item scores in the selected subscales of both questionnaires to *Z* scores and then summed each of the individual item (standardized) scores to produce a composite stress score for each timepoint.

#### Cognitive measures

Cognitive ability was examined with two tasks from the computerized CANTAB battery (Robbins et al., 1994): spatial working memory task and Cambridge gambling task. These cognitive measures were selected because they have been strongly linked to the functions of the brain regions involved in higher cognitive processes, e.g., prefrontal regions (Funahashi, 2017; Yazdi et al., 2019), and are impacted by stress exposure (Oltean, Șoflău, Miu, & Szentágotai‐Tătar, 2022; Olver, Pinney, Maruff, & Norman, 2015). In the spatial working memory task, participants were asked to search for a hidden blue token in a number of colored boxes displayed on the screen and to open the boxes to see what is inside. Using an elimination strategy, participants were asked to avoid a box where a token had previously been found. Strategy in spatial working memory task corresponds to a sequential search pattern used by the participants when beginning a new search after a token has been found. An efficient strategy requires participants to begin from a predetermined search sequence, beginning with a specific box and return to it after a token has been found (Owen, Downes, Sahakian, Polkey, & Robbins, 1990). The task progressively increases in difficulty as the number of boxes presented on the screen increases from two to eight boxes. The outcomes in this task are *between errors* which is the number of times participants revisit a box in which the token has previously been found, and *strategy* score which is number of distinct boxes used by the participants when beginning a new search. Higher scores in both of these outcomes indicate poor working memory ability. However, we reverse coded the scores, so that higher scores indicate high working memory ability.

In the Cambridge gambling task, which measures decision‐making and risk‐taking behavior, participants were presented with 10 boxes, varying in red or blue colors. Participants were told that one of the boxes contained a hidden yellow token and must first use the appropriate red or blue buttons to choose which box, they think contained the yellow token. Then, beginning with a starting 100 points, all participants will then wager an amount from a range of five possible values (5%, 25%, 50%, 75%, and 95% – sometimes presented in ascending or descending order) based on their confidence of the location of a token. If they made the correct decision, the amount will be added to their total points; otherwise, the equivalent value wagered will be deducted from their points. The outcomes analyzed in this study include *quality of decision‐making* (proportion of trials where the correct color outcome was made; higher score indicates good decision‐making ability), *risk taking* (proportion of bet points placed after the most likely outcome was chosen; higher score represents high‐risk tolerance), and *risk adjustment* (the extent to which betting behavior is moderated by the ratio of blue to red boxes presented; higher score in risk adjustment indicates that participants change total points wagered on each trials depending on the probability of winning).

#### Structural magnetic resonance imaging

Structural scans were performed on 3T scanners from different manufacturers (Philips, General Electric, Siemens, Bruker; Schumann et al., 2010). High‐resolution three‐dimensional T1‐weighted images were acquired using magnetization prepared gradient echo (MPRAGE) sequence, based on the ADNI protocol (http://www.loni.ucla.edu/ADNI/Cores/index.shtml). To account for variations in scanner manufacturer across sites, scanning protocol parameters (e.g., those relating to image contrast or signal‐to‐noise ratio) were devised and harmonized across all the scanning sites (See Appendix S2 for additional details on MRI harmonization across sites and for other quality control procedures). Scanning site was also included as a covariate in the statistical analyses. Data segmentation of structural MRI was performed using FreeSurfer version 5.3.0 (http://surfer.nmr.mgh.harvard.edu). For additional information on MRI acquisition protocols and quality control in this IMAGEN cohort (see Schumann et al., 2010). In this study, we analyzed 11 derived brain regions of interest, primarily in the frontoparietal regions in both the left and the right hemispheres; specifically, we examined the volume, surface area, and cortical thickness in the following regions of interest: middle frontal, orbitofrontal, anterior cingulate, posterior cingulate, superior frontal, frontal pole, and inferior frontal, superior parietal, inferior parietal, precuneus, and insula (See Table S1). The choice of these regions of interest was motivated by past cross‐sectional research finding that stress exposure is commonly related to differences in these brain areas (McLaughlin et al., 2019; Sheridan & McLaughlin, 2014). To reduce the number of analyses and tests conducted, we focused on these regions of interest that have been previously linked to stress, rather than analyze all potential brain areas output by Freesurfer.

### Statistical analyses

All statistical analyses were performed in *R* (R Core Team, 2016) and *R Studio* version 4.1.1, within a structural equation modeling framework (Iacobucci et al., 2007). Data for all study constructs were transformed to standardized scores to have a mean center of zero.

As a requirement for longitudinal analysis (e.g., latent change score model), we began by testing the longitudinal measurement invariance using configural, metric, and scalar models. Measurement invariance generally tests whether the constructs measured (e.g., life events questionnaire) were the same across the three different measurement waves. For each measurement invariance models tested, we first examine fit using the comparative fit index (CFI), the root mean square error of approximation (RMSEA), and standardized root mean square residual (SRMR). Previous studies (Browne & Cudeck, 1993; Hu & Bentler, 1999) have recommended the following threshold as indicative of good model fit: CFI >0.90 and RMSEA <0.08. Second, using likelihood ratio test, we then proceed to examine metric invariance by comparing the configural model against the metric model and scalar invariance by comparing metric model against the scalar model. An insignificant chi‐square difference test is an indication of measurement invariance in the construct. In addition to the chi‐square difference test, the following fit difference cutoffs have also been suggested as an indication of measurement invariance: ΔCFI >−0.010, ΔRMSEA <0.015, and ΔSRMR <0.030 (or ΔSRMR <0.010 for scalar invariance; Chen, 2007; Cheung & Rensvold, 2002; Murray, Speyer, Hall, Valdebenito, & Hughes, 2021).

**Figure 1:**
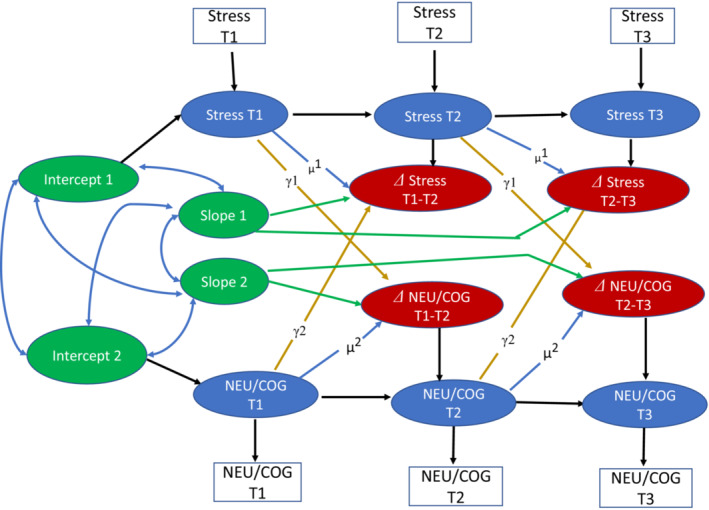
A simplified bivariate latent change score model (*N* = 502) for stress and brain metrics/cognition. We examined four bivariate relationships: 1 = relations between stress and brain volumes of frontoparietal regions; 2 = relations between stress and surface area of frontoparietal regions; 3 = relations between stress and cortical thickness of frontoparietal regions; 4 = relations between stress and cognitive outcomes. In all models, we covaried for age, sex, and sites of recruitment. T1 = Time 1; T2 = Time 2; T3 = Time 3. NEU = each frontoparietal region tested; COG = each cognitive outcome tested [Color figure can be viewed at wileyonlinelibrary.com]

To examine how individual differences (and changes) in stress exposure, brain development, and cognitive outcomes may influence one another, we first fit a bivariate latent change score model (Kievit et al., 2018; Wiedemann, Thew, Kosir, & Ehlers, 2021). In this latent change score models, depicted in Figure 1, we examined four different bivariate relations across three waves: (1) the association between stress and brain volumes in the regions of interest, (2) the relations between stress and surface area in the regions of interest; (3) the association between stress and cortical thickness in the regions of interest, and (4) the relations between stress and different cognitive outcomes in the spatial working memory and Cambridge gambling tasks. The essential feature of a latent change score model is that it can be used to test for (linear) increases or decreases within the same construct in two adjacent waves. The change score was obtained by regressing the observable score of a given timepoint from the previous timepoint (e.g., Δstress in T1–T2 or Δstress in T2–T3, where T1 = Time 1, T2 = Time 2, and T3 = Time 3). In addition, we also used cross‐lagged dynamic coupling (i.e., bidirectionality) to test for individual differences in the relations between stress and linear changes in brain/cognition as well as the relations between brain/cognition and linear change in stress. In this instance, we examined whether exposure to stress at T1 predicted a linear change in brain/cognitive scores across T1–T3 or alternatively, whether brain/cognitive scores at T1 predicted a linear change in stress across T1–T3. In total, we carried out 71 separate analyses of bivariate latent change score models examining association between stress and 11 brain volume metrics in each left and right hemisphere, 11 surface areas metrics in each left and right hemisphere, 11 cortical thickness metrics in each left and right hemisphere, and 5 cognitive outcomes. In all models, we controlled for age, sex, and sites of recruitment by adding them as covariates in the model. Of note, global brain metrics (e.g., total brain volume) were not included as covariates in our primary models. This choice was made to reduce model complexity and increase model convergence. We, however, conducted sensitivity analyses, where the total gray matter volume was entered as covariate in the model and results were fairly consistent with the results reported in our primary models. In implementing the latent change score model in *lavaan* (Rosseel, 2012), we used the maximum likelihood estimator. In each bivariate relations, we examine all model fit indices and report the parameter estimates at .05 significant threshold (uncorrected). We then applied Benjamini–Hochberg correction approach (Benjamini & Hochberg, 1995) to correct for multiple comparisons.

**Figure 2:**
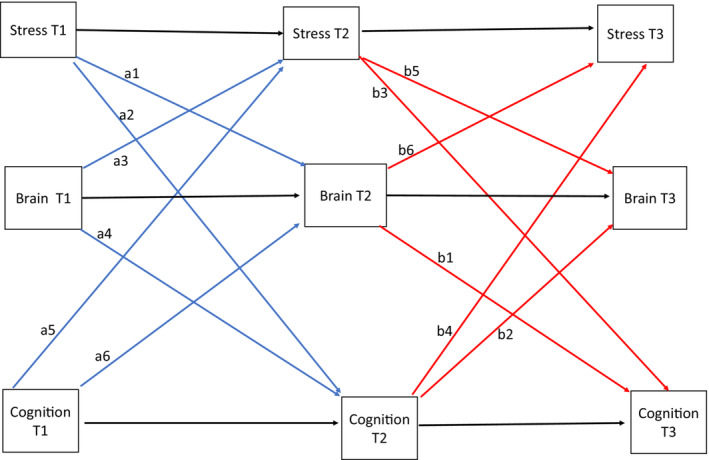
Complete longitudinal mediation model. This model investigates six possible indirect effects involving stress, brain, and cognitive variables. However, only the first two mediation paths shown in this figure were of interest in the current study. Scores at T2 and T3 are residualized [Color figure can be viewed at wileyonlinelibrary.com]

Second, using a complete longitudinal mediation model (Jose, 2016), we examine the long‐term mediating effects between stress, brain development, and cognitive functioning via autoregressive path models. A complete longitudinal mediation model (shown in Figure 2) essentially tests six different indirect paths involving all three study constructs. However, only two mediating paths that directly address our research questions are of interest in the current study: stress T1 => brain development T2 => cognitive outcome T3 and stress T1 => cognitive outcome T2 => brain development T3. Note that all scores at T2 and T3 are residualized. In the first indirect path, the model tests whether stress score at T1 predicts brain morphometrics at T2 and whether this in turn predicts the cognitive score at T3. In the second indirect path, we reversed the mediator and the outcome and examine if stress at T1 predicts the cognitive score at T2, which predicts brain morphometrics at T3. In the mediation model, we used the sum of the brain metrics in both the left and the right hemisphere rather than analyze each hemisphere separately. In total, we carried out 165 separate mediation model analyses involving 5 cognitive outcomes and the 11 brain regions of interest testing brain volume, surface area, and cortical thickness for each brain area of interest. In implementing this model in R, we completed bootstrapped estimation of the indirect effects using bias‐corrected confidence intervals at 5000 bootstrap permutations. We report the estimates of the effect sizes with their standard errors and the confidence intervals. Confidence intervals that do not include zero indicate significant indirect effects (Tan & Tan, 2010).

## Results

### Longitudinal measurement invariance

We present in Table S2 the results of the longitudinal measurement invariance, comparing configural, metric, and scalar models of our study measures. As evidence of measurement invariance, we used a nonstatistical chi‐square test and fit difference within the recommended cutoff: ΔCFI >−0.010, ΔRMSEA <0.015, and ΔSRMR <0.030 (or ΔSRMR <0.010 for scalar invariance). As shown in Table S2, the model fit of the bullying and life events questionnaires suffered from relatively poor CFI values, while other fit indices were within excellent range. The relatively poor CFI values in these questionnaires, particularly in the life events questionnaire, may be attributable to the established low inter‐item correlation (Newcomb et al., 1986). In addition, ΔCFI in both the subscales of life events questionnaire and the bullying questionnaires was outside the recommended cutoff for the metric model, indicating a lack of metric invariance in all the stress subscales. However, the scalar invariance held in the bullying questionnaire based on a nonsignificant chi‐square test and fit difference within predefined cutoffs while scalar invariance held in all the subscales of life events questionnaire based on nonsignificant chi‐square test.

On the other hand, the baseline models fit well for both the CANTAB spatial working memory and Cambridge gambling tasks. The addition of metric constraints was associated with a nonsignificant chi‐square result in the spatial working memory task but not in the Cambridge gambling task; however, ΔRMSEA increased beyond the predefined limit for the spatial working memory, while all increases and decreases were within predefined limits for the Cambridge gambling task. Scalar invariance held in both spatial working memory and Cambridge gambling tasks based on a nonsignificant chi‐square tests and fit difference well within predefined cutoffs. Taken together, while we impose scalar invariance for bullying questionnaire, and the CANTAB measures, findings in the current study should generally be interpreted with caution given the lack of clear measurement invariance in the life events questionnaire.

### Descriptive results

Results of basic demographic variables are displayed in Table 1. Shown in Table S3 is the descriptive statistics of cognitive outcomes and the summed brain metrics in the left and right hemispheres of the brain volume, surface area, and cortical thickness of the selected regions. We present the unstandardized mean score and standard deviation of these measures. We also present in Table S4, zero‐order correlations of a paired‐wave association between stress and brain/cognitive outcomes at each measurement wave as well as paired‐wave association between change in stress and brain/cognitive outcomes across adjacent waves.

**Table 1:** Mean and standard deviation (*SD*) of basic demographic variables

Variables	Mean (*SD*)	Mean (*SD*)	Mean (*SD*)
	Wave 1	Wave 2	Wave 3
Age	13.978 (0.518)	18.483 (0.725)	21.945 (0.610)
Sex	M = 219; F = 283	M = 219; F = 283	M = 219; F = 283
Sites (*N*)	Berlin = 57 Dresden = 104 London = 123 Mannheim = 114 Paris = 104		

*SD*, Standard deviation; M, male; F, female; *N*, number of participants.

### Relations between stress and brain volumes

To examine the association between stress and brain volumes in the regions of interest, we fit a bivariate latent change score model. After controlling for age, sex, and sites of recruitment, all models were within the range of excellent fit (fit range: CFI: 0.992–0.964; RMSEA: 0.053–0.071; SRMR: 0.026–0.031) as recommended in previous studies (Chen, 2007; Cheung & Rensvold, 2002). We displayed in Table 2 the lagged effects of stress on changes in each brain volume and the lagged effects of each brain volume on changes in stress.

**Table 2:** Bivariate latent change score model examining the relations between adolescent stress exposure and brain volume

	Left hemisphere		Right hemisphere	
	Lagged effects of stress	Lagged effects of brain volume	Lagged effects of stress	Lagged effects of brain volume
	γ1	γ2	γ1	γ2
	Std. *β* (*SE*)	Std. *β* (*SE*)	Std. *β* (*SE*)	Std. *β* (*SE*)
Middle frontal	−0.660 (0.494)	0.048 (0.211)	−0.221 (0.221)	0.096 (0.185)
Orbital frontal	−0.347 (0.349)	0.052 (0.209)	0.115 (0.230)	0.060 (0.187)
Anterior cingulate	−0.394 (0.480)	0.096 (0.390)	−0.327 (0.161)*	0.099 (0.222)
Inferior frontal	−0.121 (0.180)	0.149 (0.225)	−0.290 (0.245)	0.158 (0.245)
Frontal pole	0.222 (0.552)	−0.046 (0.520)	−0.427 (0.479)	−0.234 (0.427)
Superior parietal	−0.347 (0.517)	0.286 (0.495)	−0.423 (0.344)	0.257 (0.340)
Superior frontal	−0.328 (0.196)	0.010 (0.194)	−0.417 (0.694)	0.074 (0.212)
Inferior parietal	−0.636 (0.435)	0.271 (0.401)	−0.196 (0.294)	−0.065 (0.332)
Precuneus	0.123 (0.364)	0.282 (0.365)	0.264 (0.488)	−0.017 (0.364)
Posterior cingulate	−0.043 (0.167)	0.492 (0.394)	−0.285 (0.287)	0.156 (0.388)
Insula	0.097 (0.212)	0.180 (0.368)	0.049 (0.233)	−0.252 (0.405)

*SE*, Standard error.

* Significant at *p* < .05.

#### Lagged effects of stress on changes in the brain volumes

**Figure 3:**
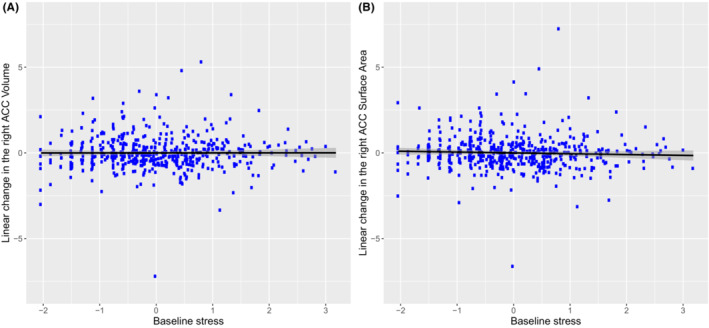
The association between baseline stress level at age 14 and linear change in the right anterior cingulate volume (Panel A) and right anterior cingulate surface area (Panel B) across ages 14–22. The bivariate latent change score analysis (*N* = 502) revealed that greater baseline stress level at age 14 was associated with a very modest reduction in the right anterior cingulate volume (Std. *β =* −.327, *p* = .042, 95% CI [−0.643, −0.012]) and right anterior cingulate surface area (Std. *β =* −.274, *p* = .038, 95% CI [−0.533, −0.015]). However, these associations became nonsignificant after correcting for multiple comparisons [Color figure can be viewed at wileyonlinelibrary.com]

First, we examined if baseline stress scores at age 14 predicted volumetric changes in regions of interest across ages 14–22. Results (displayed in Figure 3 and Table 2) showed that greater baseline adolescent stress exposure at age 14 was associated with a modest, but significant linear decrease in the right anterior cingulate volume across ages 14–22 (Std. *β =* −.327, *p* = .042, 95% CI [−0.643, −0.012]). However, this effect became nonsignificant after correcting for multiple comparisons. No other significant associations between stress and brain volumes in the other regions of interest were observed in the left hemisphere (all *p*s > .096) or in the right hemisphere (all *p*s > .219).

#### Lagged effects of brain volumes on changes in stress

Next, we investigated if baseline brain volumes at age 14 were associated with linear change in stress exposure reported across ages 14–22. We found no statistically significant evidence of lagged effects of brain volumes on changes in reported stress exposure in the left hemisphere (all *p*s > .213) or in the right hemisphere (all *p*s > .451).

### Relations between stress and brain surface area

Paralleling the approach above, we next examined relations between individual differences in change between stress exposure and surface area of brain regions of interest. Examination of model fit showed that all models were within the range of excellent fit (fit range: CFI: 0.994–0.981; RMSEA: 0.051–0.073; SRMR: 0.026–0.030). Results presented in Table 3 displayed the lagged effects of stress on changes in surface area of each brain regions and the lagged effects of brain surface area on stress.

**Table 3:** Bivariate latent change score model examining the relations between adolescent stress exposure and brain surface area

	Left hemisphere		Right hemisphere	
	Lagged effects of stress	Lagged effects of brain surface area	Lagged effects of stress	Lagged effects of brain surface area
	γ1	γ2	γ1	γ2
	Std. *β* (*SE*)	Std. *β* (*SE*)	Std. *β* (*SE*)	Std. *β* (*SE*)
Middle frontal	−.366 (0.206)	.194 (0.161)	−.176 (0.141)	.309 (0.189)
Orbital frontal	−.571 (0.307)	.158 (0.175)	−.061 (0.041)	.087 (0.125)
Anterior cingulate	−.144 (0.198)	.278 (0.357)	−.274 (0.132)*	.366 (0.276)
Inferior frontal	.095 (0.151)	.212 (0.197)	−.121 (0.185)	.313 (0.266)
Frontal pole	.766 (0.596)	.573 (0.888)	.173 (0.307)	.144 (0.461)
Superior parietal	−.217 (0.460)	.156 (0.365)	−.243 (0.345)	.209 (0.314)
Superior frontal	−.197 (0.104)	.260 (0.177)	−.163 (0.189)	.341 (0.257)
Inferior parietal	−.318 (0.277)	.290 (0.348)	−.075 (0.248)	−.107 (0.440)
Precuneus	.221 (0.388)	.257 (0.411)	−.161 (0.489)	.067 (0.342)
Posterior cingulate	−.002 (0.186)	.467 (0.280)	.035 (0.236)	.746 (0.697)
Insula	.042 (0.434)	−.426 (1.249)	.022 (0.453)	−.335 (0.662)

*SE*, Standard error.

* Significant at *p* < .05.

#### Lagged effects of stress on changes in the brain surface area

First, we examined if baseline stress exposure at age 14 was associated with linear changes in the surface area of regions of the interest across ages 14–22. Results (displayed in Figure 3 and Table 3) showed that baseline greater stress exposure at age 14 was associated with a modest, but significant linear decrease in the right anterior cingulate surface area across ages 14–22 (Std. *β =* −.274, *p* = .038, 95% CI [−0.533, −0.015]). As was the case with brain volume, this significant effect did not hold after correcting for multiple comparisons. No other significant associations between stress and cortical surface area in regions of interest were observed in the left hemisphere (all *p*s > .059) or in the right hemisphere (all *p*s > .138).

#### Lagged effects of surface area at baseline on changes in stress

Similarly, we examined if baseline brain surface area at age 14 was associated with linear change in stress exposure reported across ages 14–22. We found no evidence of significant associations between age 14 surface area in the selected brain regions and linear change in stress exposure reported across ages 14–22. This was true for both the left hemisphere (all *p*s > .096) and the right hemisphere (all *p*s > .104).

### Relations between stress and brain cortical thickness

We next examined the relationship between stress and cortical thickness in each region of interest. Inspection of model fit revealed that all models were within the range of acceptable fit (fit range: CFI: 0.984–0.939; RMSEA: 0.054–0.084; SRMR: 0.027–0.033). We followed a similar strategy used in the two bivariate relations described above by examining the lagged effects of baseline stress on changes in cortical thickness and the lagged effects of cortical thickness on stress exposure (shown in Table 4).

**Table 4:** Bivariate latent change score model of adolescent stress and brain cortical thickness

	Left hemisphere		Right hemisphere	
	Lagged effects of stress	Lagged effects of cortical thickness	Lagged effects of stress	Lagged effects of cortical thickness
	γ1	γ2	γ1	γ2
	Std. *β* (*SE*)	Std. *β* (*SE*)	Std. *β* (*SE*)	Std. *β* (*SE*)
Middle frontal	−.350 (0.349)	−.089 (0.157)	.235 (0.268)	−.130 (0.186)
Orbital frontal	.328 (0.334)	−.752 (0.449)	.281 (0.933)	−.338 (0.225)
Anterior cingulate	.060 (0.044)	−.022 (0.073)	.021 (0.306)	−.175 (0.191)
Inferior frontal	−.333 (0.295)	.490 (0.890)	−.207 (0.506)	−.061 (0.299)
Frontal pole	−.186 (0.423)	−.516 (0.459)	−.565 (0.524)	.064 (0.346)
Superior parietal	−.275 (0.530)	.194 (0.278)	−.181 (0.320)	.157 (0.206)
Superior frontal	−.162 (0.239)	−.125 (0.144)	.110 (0.591)	.110 (0.591)
Inferior parietal	−.590 (0.634)	.140 (0.314)	−.122 (0.442)	.230 (0.239)
Precuneus	−.058 (0.402)	.015 (0.318)	.115 (0.260)	−.020 (0.181)
Posterior cingulate	−.361 (0.461)	−.685 (0.605)	.034 (0.035)	.007 (0.066)
Insula	−.288 (0.673)	.738 (0.699)	−.780 (0.776)	−.030 (0.488)

*SE*, standard error.

#### Lagged effects of stress on changes in cortical thickness

We then examined whether baseline stress at age 14 was associated with linear increases or decreases in cortical thickness across ages 14–22. We found no evidence of significant association between baseline stress level at age 14 and linear change in cortical thickness across ages 14–22 in both left (*p* > .168) and right (*p* > .281) hemispheres.

#### Lagged effects of cortical thickness on changes in stress

Next, we examined whether baseline cortical thickness at age 14 was associated with linear change in stress across ages 14–22. Results showed that there was no statistically significant association between brain cortical thickness in any of the regions of interest examined at age 14 and linear changes in stress exposure reported across ages 14–22. This was true for both the left hemisphere (all *p*s > .095) and the right hemisphere (all *p*s > .134).

### Relations between stress and different cognitive outcomes

The last bivariate association we examined was the relation between stress and different cognitive outcomes in the spatial working memory and Cambridge gambling tasks. The cognitive variables tested include strategy and between error scores in the spatial working memory task, as well as quality of decision‐making, risk taking, and risk adjustment in the Cambridge gambling task. Model inspection showed that all models fit well (fit range: CFI 0.978–0.960; RMSEA 0.061–0.067; SRMR 0.030–0.029). Results shown in Table 5 revealed that there was no significant association between baseline stress level at age 14 and linear changes in any of the cognitive outcome across ages 14–22 (all *p*s > .185), or vice versa (all *p*s > .110).

**Table 5:** Bivariate latent change score model of adolescent stress and cognitive outcomes

	Lagged effects of stress	Lagged effects of cognition
	γ1	γ2
	Std. *β* (*SE*)	Std. *β* (*SE*)
SWM strategy	−.719 (0.681)	.042 (0.529)
SWM errors	.007 (0.042)	.086 (0.054)
CGT quality of decision‐making	−.772 (0.581)	−.161 (0.364)
CGT risk taking	.227 (0.880)	.750 (0.581)
CGT risk adjustment	−.334 (0.349)	−.192 (0.525)

*SE*, standard error.

### Mediation analysis

Lastly, through autoregressive path models, we completed longitudinal mediation analyses of stress, brain metrics, and cognitive outcomes. Depicted in Figure 2, there are potentially six indirect paths involving these three variables. We, however, report only two paths relevant to our research questions. In the first of these two indirect path analyses, we examined if stress scores at Time 1 predict residualized brain scores at Time 2 and if this in turn predicts cognitive performance at Time 3. Results presented in Table S5 showed that no significant indirect associations were observed for this first indirect path involving adolescent stress, brain variables (volume, surface area, and cortical thickness), and cognitive outcomes. The confidence intervals of each of these mediation models included zero, indicating that they were not statistically significant. Similarly, we examined a second indirect path of effects, by reversing the mediators and outcomes. This second model examined if stress at Time 1 predicts cognitive performance at Time 2 which in turn predicts brain volume, surface area, or cortical thickness at Time 3. Similar to the first set of mediation analyses, we found no evidence of any indirect association involving this path, as the confidence intervals in each mediation model included zero, indicating a lack of significant indirect effects.

### Sensitivity analyses

We reanalyzed the bivariate latent change models that examined the longitudinal association between stress and cortical structures by including the total gray matter volume as a covariate in the model. Results of the sensitivity analyses showed that the associations did not substantially vary from the results of the primary analyses that did not include the total gray matter volume as covariate. With the exception of left superior frontal volume which showed significant reduction across ages 14–22 as a result of baseline stress at age 14, no other inconsistency was observed between the sensitivity analyses and the primary analyses. As was the case with primary analyses, no result in the sensitivity analyses survived correction for multiple comparisons. Results of the sensitivity analyses are shown in Table S6 for model examining stress and brain volume, Table S7 for model examining cortical surface area, and Table S8 for model examining cortical thickness.

## Discussion

In a three‐wave longitudinal cohort sample, this study used a latent change score model to examine the bivariate relations between stress and the trajectories of three brain morphometric measures (i.e., volume, surface area, and cortical thickness) in selected regions of interest. Using similar analytic approaches, we also investigated the association between stress exposure and various cognitive outcomes. This analytic strategy allows us to test the bidirectional effects of individual differences in change in the relations between stress, brain development, and cognitive outcomes. As such, our work can help us understand if stress predicts changes in outcomes and/or if these outcomes predict change in stress. After controlling for age, sex, and sites of recruitment, results showed that baseline stress levels at age 14 predicted a small decrease in the volume and surface area of the right anterior cingulate cortex across ages 14–22. We found no evidence of significant relations between stress and cortical thickness in the selected brain regions or cognitive outcomes. In a separate, connected analysis, we used longitudinal mediation models to further explore interrelations between stress, brain morphometrics, and cognitive outcomes, exploring two potential indirect paths – indirect effects of brain morphometrics and indirect effects of cognitive outcomes. Statistical models showed no evidence of indirect effects in any of the two indirect paths that we examined.

Examining our findings, there are a number of important connections to note related to previous studies. First, we found evidence of lagged effects of stress on the volume and surface area of the right anterior cingulate. We must caution that these associations are very modest in nature, and after correcting for multiple comparisons, these associations became nonsignificant. Connecting these findings to previous studies, due to its protracted neurodevelopment, portions of prefrontal cortex, including the anterior cingulate, are presumed to be more vulnerable to adversity exposure in childhood (Arnsten, 2009; Lupien et al., 2009). Supporting this assumption, previous studies investigating prefrontal regions have shown some of the most consistent structural reductions following stress compared to many other brain regions (Gold et al., 2016; Hodel et al., 2015; Mackes et al., 2020; McLaughlin et al., 2014). The smaller right anterior cingulate volume and surface area, observed in our study, are also consistent with a few other cross‐sectional studies that have found smaller anterior cingulate volume following stress (Ansell, Rando, Tuit, Guarnaccia, & Sinha, 2012; Hanson et al., 2012; Hodel et al., 2015; Jensen et al., 2015). These reductions in prefrontal volumes linked to stress have been found to be driven by differences in the brain surface area rather than the cortical thickness (Hodel et al., 2015), providing further evidence of structural and cellular differences in these brain morphometrics and why they may have varying level of vulnerability to stress. This may explain why we found smaller right anterior cingulate volume and surface area associated with stress but not in the cortical thickness, suggesting that the effects of stress on brain volume may be more approximate to the effects of stress on cortical surface area than on cortical thickness. However, the modest nature of these effects in our study, which became nonsignificant after correcting for multiple comparisons, suggests that the long‐term effects of stress on cortical structures, including in prefrontal regions, may not be as large as have been suggested in previous cross‐sectional studies.

At a broad level, we noted only modest associations between stress exposure and the three brain morphometrics measured in our study. This stands in stark contrast with the widespread reductions in frontoparietal regions reported elsewhere (Baker et al., 2012; Cohen et al., 2006; Gold et al., 2016; Hodel et al., 2015; Kelly et al., 2013; Kennedy et al., 2021; Mackes et al., 2020; McLaughlin et al., 2014; Teicher, 2006; Thomaes et al., 2010). There are multiple explanations for these potential discrepancies. First, most of these past studies recruited participants who have been exposed to extreme forms of adversity (e.g., institutionalization and maltreatment). These types of extreme stress or adversity have been linked to higher likelihood of developing psychopathology. This is in contrast to our study, which examined a general population, representative cohort. It is likely that the stress exposure that we measured here (i.e., general lower level environmental and family stressors) do not exert as much impact on brain development, as the more extreme adversities examined in past published reports. An alternative explanation to the small, nonsignificant effects observed in our study may also be related to the longitudinal modeling used in our study. Our approach was designed to capture increases or decreases present in the bivariate and bidirectional relations between stress and the brain morphometrics. As such changes are often based on auto‐regressive assumptions (i.e., differences in scores between two timepoints), significant effects may be less likely to be observed compared to the robust reductions in cortical structures previously reported in cross‐sectional studies. As noted in our supporting information and supporting this point, results of our paired‐wave correlational analysis between stress exposure and the brain morphometrics showed widespread significant associations. A third possible explanation may be the developmental timing of stress in our study. Although the study asked about the lifetime exposure to stress at 14, later stress measurements at ages 19 and 22 primarily centered on stressful events that had happened since the time of a participant's last study visit. Previous work on the sensitive periods of adversity (Dunn et al., 2019; Marini et al., 2020; Nweze et al., 2022) has observed that the effects of adversity on outcomes are greater when exposures occur in very early childhood (than in later childhood). Past cross‐sectional studies on brain development and adversity have commonly focused on samples with stress exposure in the very early years of life (i.e., institutionalization at ages 1–3). This idea is further strengthened by previous work in a community‐based sample (Birn et al., 2017). A functional imaging study findings showed that those with high levels of childhood stress had less brain activations, including in many frontoparietal regions, during an fMRI task designed to measure risk taking and decision‐making relative to a comparison group that was lower on stress exposure levels. More importantly, the effects observed in their study were driven by childhood stress, rather than current (adult) stress level. Another recent study (Gehred et al., 2021) showed that both prospectively and retrospectively ascertained childhood stresses were associated with reductions in the structural integrity of the brain; however, the prospectively ascertained childhood stress had larger and widely distributed effects on the brain compared to retrospective childhood stress reported in adulthood. Taken together, these past studies collectively suggest that the intensity of stress and also the developmental time of stress exposure may explain the limited effects observed in our study.

Additionally, by simultaneously examining three brain morphometric measures, we probed if adversity has equally distributed or disproportionate effects on brain volume, surface area, and cortical thickness. Based on the findings observed in our study, the answer to this question is, presently, inconclusive. We observed no bivariate relationship between brain cortical thickness and stress and reported lagged effects of stress on the volume and surface area of the right anterior cingulate. However, the very small nature of these effects which became nonsignificant after correcting for multiple comparisons does not convince us to make any concrete conclusion in this regard. Almost all previous studies have examined these three brain morphometrics separately in relation to how they are altered by stress. The only studies that have simultaneously examined at least two of these three morphometrics have reported equally distributed reductions in cortical surface area and thickness (Gehred et al., 2021), regional specific reductions (e.g., smaller right inferior frontal volume and surface area; Mackes et al., 2020), or no reductions at all (Rinne‐Albers et al., 2020). More studies are needed before we can concretely ascertain the extent of widespread changes or reductions in all three brain morphometrics following adversity.

Lastly, we examined if complete longitudinal mediation models can provide insight into the neurocognitive pathways potentially mediating the interrelations between stress, brain, and cognitive outcomes. In doing so, we examine two crucial indirect paths – first, the path from which the effects of stress on brain morphometrics at earlier assessments reflects on cognitive functioning later on; and an alternative path where the effects of stress on cognition at an earlier wave impacts the brain development at the later assessment. We found no evidence of indirect association in any of the two indirect paths we reported. While stress is known to predict reductions in the prefrontal cortex and cognitive abilities, we have no knowledge of studies that have examined their mediating effects. And most knowledge about their interrelations come from functional imaging studies. Additional work is needed to further confirm the absence of neurocognitive mediating effects. Possible reasons for the lack of indirect effects observed may mirror explanations for limited effects observed in the bivariate latent change analysis. For example, all brain and cognitive scores at timepoint 2 and timepoint 3 were residualized, and as noted by Jose (2016), such highly auto‐regressive paths of mediation analysis would ordinarily diminish any potential indirect effects present in the data. We encourage future studies that would similarly probe this longitudinal mediating effect.

### Limitations of the study

Our study has a number of notable strengths, including the use of a longitudinal design in a large cohort sample. However, the work is not without limitations, especially related to our measurement of stress. First, the stress questionnaire used in the study provided important insights into deviations from the expected childhood environment; however, these measures focus on experiences that are of a lesser degree of severity than some adversity measures used previously in the literature (e.g., child institutionalization and maltreatment). Our study would have been enhanced by the use of some of these severe adversity measures. Second, given the importance of timing of exposure in our understanding of effects of adversity (Dunn et al., 2019), our study would have benefitted from stress data that was prospectively captured across early childhood development. Third, it should be noted that the total brain volume, surface area, and cortical thickness were not controlled in our study, and this should be taken into perspective when interpreting the reported results. Lastly, although all subscales of the bullying and life events questionnaires used for our measurement of stress exposure were within the cutoffs of metric or scalar measurement invariance (See Table S2), the CFI fit of these subscales suffered considerably from poor values. Possible reasons for the relatively poor CFI values observed in the stress measures may be down to the nuances in the measurement protocols. For example, the life events questionnaire at baseline (age 14) was based on lifetime retrospective reports where the participants were asked if they had been exposed to a particular negative event and to indicate the age or time of exposure. Subsequent questions at follow‐up assessments were framed to reflect experiences that had occurred after the baseline measurement and in‐between each follow‐up assessment. This retrospective responding (provided at baseline) may have biased the model fit of measurement invariance analysis in a substantial way. Alternatively, the low CFI scores may be due to the low inter‐item correlation noted for the life event questionnaire (Newcomb et al., 1981), especially given that all other fit indices for these stress measures were within excellent range. Lastly, it should be noted that only baseline stress measure was associated with smaller right anterior cingulate volume and surface area; and as such, the lack of measurement invariance is less problematic. However, the lack of effect observed between longitudinal stress measurement and baseline cortical structures may be due to lack of invariance in stress measures, rather than a lack of true effects between longitudinal stress and baseline cortical structures. Given the limitations of the study highlighted above, we urge the readers to interpret the findings of the current study with caution.

## Conclusion

We found that baseline stress levels were associated with a decrease in the right anterior cingulate volume and surface area. Both of these effects were very small and became nonsignificant after correcting for multiple comparisons. Findings shed light on the impact of stress on structural brain reductions, particularly in the prefrontal cortex that have consistently been implicated in the previous cross‐sectional studies. Examined collectively, our study to some extent suggests that the effects of stress on cortical structures and cognitive functioning may not be as robust as have been suggested in previous cross‐sectional projects.

## Funding

T.N. received funding from the Cambridge Trust (University of Cambridge).

## Supporting information


**Appendix S1.** Study questionnaires and subscales.
**Appendix S2.** MRI harmonization across sites and quality control procedures.
**Table S1.** Selected brain regions of interest and their corresponding parcel names.
**Table S2.** Model fits of longitudinal invariance testing.
**Table S3.** Mean and standard deviation (*SD*) of cognitive and brain data across the three waves.
**Table S4.** A zero‐order correlation of a paired‐wave association between adolescent stress exposure and brain/cognitive outcomes as well as association between change in stress and change in brain/cognitive outcomes.
**Table S5.** Complete longitudinal mediation analyses showing indirect effects of different brain regions (path 1) or cognitive functioning (path 2) in the relations between adolescent stress exposure, brain development and cognitive functioning.
**Table S6.** Bivariate latent change score model examining the relations between adolescent stress exposure and brain volume, controlling for Total Gray Matter volume.
**Table S7.** Bivariate latent change score model examining the relations between adolescent stress exposure and brain surface area, controlling for Total Gray Matter volume.
**Table S8.** Bivariate latent change score model of adolescent stress and brain cortical thickness, controlling for Total Gray Matter volume.
