# Supplementary material for: Trajectories of cortical structures associated with stress across adolescence: a bivariate latent change score approach
Source: J Child Psychol Psychiatry. 2023 Mar 29;64(8):1159–75. doi: 10.1111/jcpp.13793 (PMC10952720; doi:10.1111/jcpp.13793)
Supplement: Supplementary file 1 — Appendix S1. Study questionnaires and subscales. Appendix S2. MRI harmonization across sites and quality control procedures. Table S1. Selected brain regions of interest and their corresponding parcel names. Table S2. Model fits of longitudinal invariance testing. Table S3. Mean and standard deviation (SD) of cognitive and brain data across the three waves. Table S4. A zero‐order correlation of a paired‐wave association between adolescent stress exposure and brain/cognitive outcomes as well as association between change in stress and change in brain/cognitive outcomes. Table S5. Complete longitudinal mediation analyses showing indirect effects of different brain regions (path 1) or cognitive functioning (path 2) in the relations between adolescent stress exposure, brain development and cognitive functioning. Table S6. Bivariate latent change score model examining the relations between adolescent stress exposure and brain volume, controlling for Total Gray Matter volume. Table S7. Bivariate latent change score model examining the relations between adolescent stress exposure and brain surface area, controlling for Total Gray Matter volume. Table S8. Bivariate latent change score model of adolescent stress and brain cortical thickness, controlling for Total Gray Matter volume. [file JCPP-64-1159-s001.docx]

Methods

**Appendix S1: Study questionnaires and subscales**

Adversity: Life events questionnaire (Newcomb, Huba, & Bentler, 1981) and the revised Olweus bully/victim questionnaire (Olweus, 2006) were both used in operationalizing adversity. The following are the subscales and item questions used in life events questionnaire: (note that at baseline assessment, they were asked if these events ever occurred but in the two later follow-up assessments, they were asked if the events occurred since their last IMAGEN assessments).

Parents and Family subscale

1. parents divorced
2. family had money problems
3. Parents argued or fought
4. Mother/father remarried
5. Parent abused alcohol

Accident and illness subscale

1. A close family member had a severe accident or a severe illness
2. Given medication by my doctor
3. Death of a relative you are close to
4. I had a serious accident or illness (e.g. broke leg)

In bully/victim questionnaire, participants were asked how often have the following situations occurred in the past 6 months?

1. I was bullied at school (a student/ peer said or did nasty or unpleasant things to me).
2. I was called mean names, was made fun of, or teased in a hurtful way by a student/ peer.
3. A student/ peer left me out of things on purpose, excluded me from their group of friends or completely ignored me
4. I was hit, kicked, pushed or shoved around, or locked indoors by a student/ peer.
5. I have been bullied by a teacher.
6. I have been bullied by a family member.

**Appendix S2. MRI harmonization across sites and quality control procedures**

Due to scanning taking place at different sites using 3T scanners from different manufacturers, there was a need to account for variations from data acquired from different MRI scanners as a result of implementations of specific image-acquisition technique (Schumann et al., 2010). An attempt was made to harmonize all MRI data to reduce such variations. To achieve this aim, so many scanning parameters compatible to scanners across all sites were devised and held constant especially those relating to image contrast or signal-to-noise ratio. Two quality control procedures were used to do this;

1. Information about geometric distortions, image contrast, temporal stability, signal to noise to ratio, and differences in radiofrequency coils was obtained by scanning American College of Radiology phantom.
2. For information about protocol factors that cannot be obtained using phantom, healthy volunteers were regularly scanned across sites and at multiple sites to account for inter-site variability in structural measures.

Other data pre-processing quality control: As part of the quality control procedures of data pre-processing, MRI data was also visually inspected by independent raters. Structural scans with segmentation inaccuracies, weakness in normalization, clinical abnormalities, motion artefacts, susceptibility artifacts and deformation were flagged. For full details about IMAGEN study, including exclusion criteria, study protocols, MRI acquisition parameters etc., see Schumann et al., 2010.

| Table S1 | |
| --- | --- |
| Selected brain regions of interest and their corresponding parcel names | |
| Regions of interest | Parcel names |
|  |  |
| Middle frontal | Caudal middle frontal |
|  | Rostral middle frontal |
| Orbital frontal | Medial orbital frontal |
|  | Lateral orbital frontal |
| Anterior cingulate | Caudal anterior cingulate |
|  | Rostral anterior cingulate |
| Inferior frontal | Pars opercularis |
|  | Pars orbitalis |
|  | Pars triangularis |
| Frontal pole | Frontal pole |
| Superior parietal | Superior parietal |
| Superior frontal | Superior frontal |
| Inferior parietal | Inferior parietal |
| Precuneus | Precuneus |
| Posterior cingulate | Posterior cingulate |
| Insula | Insula |
| Note: The brain regions with two or more different components were summed to produce the metric for the brain region. This approach was used for the brain volume, brain surface area and cortical thickness for both the left and right hemispheres. | |

Results

**Paired-wave correlational results**

We present in Table S3, zero-order correlations of paired-wave associations between stress and brain/cognitive outcomes at each measurement wave and paired-wave associations between change in stress and change in brain/cognitive outcomes.

***Timepoint 1 (age 14):*** At age 14, we found significant negative correlations between stress and left orbital frontal cortical thickness (r = -.10, p<0.05), and left insula cortical thickness (r = -.10, p < 0.05). In the right hemisphere, we observed significant negative correlations between stress at age 14 and inferior parietal volume (r = -.12, p < 0.01), posterior cingulate volume (r = -.09, p < 0.05), inferior parietal surface area (r = -.12, p < 0.01), posterior cingulate surface area (r = -.10, p < 0.05), and insula cortical thickness ( r = -.10, p <0.05).

***Timepoint 2 (age 19):*** At age 19, we observed significant negative associations between stress and left anterior cingulate volume (r = -.15, p < 0.001) left middle frontal surface area (r = -.09, p < 0.05), left anterior cingulate surface area (r = -.15, p < 0.01), and left inferior parietal surface area (r = -.10, p < 0.05). In the same timepoint 2, we observed signification correlations between stress and the right inferior parietal volume (r = -.10, p < 0.05), right orbitofrontal surface area (r = -.10, p < 0.05), right superior frontal surface area (r = -.09, p < 0.05), right middle frontal cortical thickness (r = .10, p < 0.05), right orbital frontal cortical thickness (r = .10, p < 0.05), right inferior frontal cortical thickness (r = .09, p < 0.05), right frontal pole cortical thickness (r = 10, p < 0.05), right superior parietal cortical thickness (r = .09, p < 0.05), right superior frontal cortical thickness (r = .14, p < 0.01) and right posterior cingulate cortical thickness (r = .13, p < 0.01).

***Timepoint 3 (age 22):*** We examined significant correlations between stress and cortical structures/cognitive outcomes at age 22. In the left hemisphere, we observed significant negative associations between stress and anterior cingulate volume (r = -.11, p < 0.05), inferior parietal volume (r = -.12, p < 0.01), middle frontal surface area (r = -.09, p < 0.05), anterior cingulate surface area (r = -.10, p < 0.05), and inferior parietal surface area (r = -.11, p < 0.05). On the other hand, we found significant negative correlations between stress and right anterior cingulate volume (r = -.12, p < 0.01) and right anterior cingulate surface area (r = -.10, p < 0.05). Finally, we report significant negative association between stress and risk adjustment in the Cambridge Gambling Task (r = -.13, p < 0.01) but positive association with risk taking (r = .12, p < 0.01) in the same gambling task.

***Δstress T2-T3 and Δbrain/cognition T2-T3:*** On the other hand, we examined the correlation between change in stress and change in brain or cognitive outcomes. Change scores in these variables across waves were obtained using simple difference scoring (subtraction) approach. Result showed a significant negative correlation between change in stress and change in the left middle frontal volume (r = -.15, p <0.001), left orbital frontal volume (r = -.11, p < 0.05), left anterior cingulate volume (r = -.13, p < 0.01), right inferior parietal volume (r = -. 12, p < 0.01), left orbital frontal surface area (r = -.10, p < 0.05), left anterior cingulate cortical thickness (r = -.10, p < 0.05), left posterior cingulate cortical thickness (r = -.09, p < 0.05), and right posterior cingulate cortical thickness (r = -.10, p < 0.01). See Table S3 for all correlational results.

Other supplementary Tables

| Table S2: Model fits of longitudinal invariance testing | | | | | | | | | |
| --- | --- | --- | --- | --- | --- | --- | --- | --- | --- |
|  | Fit | | | Fit difference versus baseline | | | Δχ2 Difference test | | |
| Model | CFI | RMSEA | SRMR | ΔCFI | ΔRMSEA | ΔSRMR | Δχ2 | df | p |
| **LEQ: Parents and Family subscale** |  |  |  |  |  |  |  |  |  |
| Configural | 0.884 | 0.037 | 0.058 |  |  |  |  |  |  |
| Metric | 0.815 | 0.045 | 0.063 | -0.069 | 0.008 | 0.005 | 26.766 | 80 | <0.001 |
| Scalar | 0.828 | 0.041 | 0.064 | 0.013 | -0.004 | 0.001 | 2.869 | 88 | 0.942 |
|  |  |  |  |  |  |  |  |  |  |
| **LEQ: Accident and illness subscale** |  |  |  |  |  |  |  |  |  |
| Configural | 0.926 | 0.029 | 0.032 |  |  |  |  |  |  |
| Metric | 0.863 | 0.037 | 0.050 | -0.063 | 0.008 | 0.018 | 18.443 | 45 | 0.005 |
| Scalar | 0.845 | 0.037 | 0.052 | -0.018 | 0.000 | 0.002 | 11.530 | 51 | 0.073 |
|  |  |  |  |  |  |  |  |  |  |
| **Bullying: victim subscale** |  |  |  |  |  |  |  |  |  |
| Configural | 0.922 | 0.029 | 0.057 |  |  |  |  |  |  |
| Metric | 0.869 | 0.036 | 0.061 | -0.053 | 0.007 | 0.004 | 26.712 | 124 | 0.002 |
| Scalar | 0.868 | 0.035 | 0.062 | -0.001 | -0.001 | 0.001 | 14.577 | 134 | 0.148 |
|  |  |  |  |  |  |  |  |  |  |
| **Spatial working memory (CANTAB)** |  |  |  |  |  |  |  |  |  |
| Configural | 0.997 | 0.000 | 0.008 |  |  |  |  |  |  |
| Metric | 0.997 | 0.056 | 0.012 | 0.000 | 0.056 | 0.005 | 1.124 | 2 | 0.57 |
| Scalar | 0.999 | 0.024 | 0.012 | 0.002 | -0.032 | 0.000 | 0.000 | 4 | >0.999 |
|  |  |  |  |  |  |  |  |  |  |
| **Cambridge gambling task (CANTAB)** |  |  |  |  |  |  |  |  |  |
| Configural | 0.979 | 0.069 | 0.055 |  |  |  |  |  |  |
| Metric | 0.974 | 0.069 | 0.055 | -0.005 | 0.000 | 0.000 | 13.792 | 19 | 0.007 |
| Scalar | 0.976 | 0.060 | 0.055 | 0.002 | -0.009 | 0.000 | 0.000 | 23 | >0.999 |
| Note: CFI = Comparative Fit Index, RMSEA = Root Mean Square Error of Approximation, and SRMSR = Standardized Root Mean Square Residual, df = degrees of freedom | | | | | | | | | |

| Table S3 | | | |
| --- | --- | --- | --- |
| Mean and standard deviation (SD) of cognitive and brain data across the three waves | | | |
|  | Mean (SD) | Mean (SD) | Mean (SD) |
|  | Wave 1 | Wave 2 | Wave 3 |
|  |  |  |  |
| **CANTAB** |  |  |  |
| SWM Strategy | 33.476 (5.426) | 35.731 (5.918) | 21.195 (5.761) |
| SWM Errors | 56.067 (13.456) | 63.894 (11.369) | 102.982 (10.490) |
| CGT quality of decision | 0.945 (0.078) | 0.957 (0.069) | 0.971 (0.048) |
| CGT risk taking | 0.527 (0.140) | 0.541 (0.125) | 0.563 (0.125) |
| CGT risk adjustment | 1.683 (1.039) | 1.998 (1.019) | 2.168 (0.982) |
|  |  |  |  |
| **Brain volume (both hemispheres)** |  |  |  |
| Middle frontal | 49095.29 (8636.80) | 46570.165 (6806.821) | 45013.267 (6291.685) |
| Orbital frontal | 27285.345 (4126.635) | 26252.343 (3089.147) | 27407.12 (3062.62) |
| Anterior cingulate | 9873.498 (1913.046) | 9713.954 (1575.832) | 8969.474 (1469.633) |
| Inferior frontal | 24685.219 (3750.156) | 23441.745 (2997.008) | 23263.699 (2807.588) |
| Frontal pole | 2319.125 (419.095) | 2095.284 (357.140) | 2354.410 (379.484) |
| Superior parietal | 29106.661 (4151.615) | 27332.596 (3494.912) | 26704.992 (3383.698) |
| Superior frontal | 49848.498 (7231.121) | 47148.199 (6177.104) | 46177.255 (5948.066) |
| Inferior parietal | 31636.476 (4909.638) | 29630.645 (4054.452) | 27827.319 (3620.887) |
| Precuneus | 22503.669 (3358.433) | 21376.313 (2832.298) | 20860.968 (2662.028) |
| Posterior cingulate | 7347.494 (1170.426) | 7107.309 (1031.666) | 6865.147 (901.088) |
| Insula | 14132.253 (1800.248) | 13691.410 (1641.206) | 14894.390 (1660.356) |
|  |  |  |  |
| **Cortical surface area (both hemispheres)** |  |  |  |
| Middle frontal | 16115.715 (2636.346) | 16481.55 (2171.08) | 16243.355 (2068.934) |
| Orbital frontal | 8599.116 (1286.807) | 8773.233 (1021.791) | 9309.596 (1016.944) |
| Anterior cingulate | 2916.5478 (531.4816) | 3000.998 (483.834) | 2807.337 (457.481) |
| Inferior frontal | 7414.767 (1062.593) | 7498.197 (901.881) | 7540.035 (869.845) |
| Frontal pole | 522.677 (77.013) | 513.918 (66.408) | 590.336 (66.241) |
| Superior parietal | 11057.259 (1427.673) | 11090.339 (1229.972) | 10920.871 (1247.024) |
| Superior frontal | 14358.747 (2017.029) | 14553.024 (1721.082) | 14613.783 (1695.324) |
| Inferior parietal | 10279.618 (1499.736) | 10312.249 (1339.871) | 9965.137 (1296.762) |
| Precuneus | 7929.530 (1129.312) | 7980.468 (1021.551) | 7930.131 (997.706) |
| Posterior cingulate | 2413.233 (357.757) | 2454.163 (343.760) | 2418.719 (314.299) |
| Insula | 4153.358 (503.063) | 4149.802 (480.136) | 4701.071 (512.625) |
|  |  |  |  |
| **Cortical thickness (both hemispheres)** |  |  |  |
| Middle frontal | 10.228 (0.619) | 9.802 (0.652) | 9.752 (0.573) |
| Orbital frontal | 10.742 (0.639) | 10.359 (0.612) | 10.479 (0.513) |
| Anterior cingulate | 11.827 (0.963) | 11.464 (0.751) | 11.306 (0.672) |
| Inferior frontal | 16.387 (0.989) | 15.828 (0.864) | 15.773 (0.783) |
| Frontal pole | 5.959 (0.647) | 5.690 (0.594) | 5.518 (0.519) |
| Superior parietal | 4.618 (0.296) | 4.408 (0.275) | 4.378 (0.231) |
| Superior frontal | 5.808 (0.362) | 5.557 (0.419) | 5.439 (0.381) |
| Inferior parietal | 5.298 (0.325) | 5.072 (0.265) | 4.927 (0.222) |
| Precuneus | 5.170 (0.306) | 4.965 (0.260) | 4.920 (0.201) |
| Posterior cingulate | 5.548 (0.347) | 5.329 (0.290) | 5.124 (0.264) |
| Insula | 6.526 (0.350) | 6.410 (0.267) | 6.286 (0.243) |
| Note: SD = standard deviation; SWM = spatial working memory; CGT = Cambridge gambling task | | | |

| Table S4: A zero-order correlation of a paired-wave association between adolescent stress exposure and brain/cognitive outcomes as well as association between change in stress and change in brain/cognitive outcomes. | | | | | | | | | | |
| --- | --- | --- | --- | --- | --- | --- | --- | --- | --- | --- |
|  | Left hemisphere | | | | | Right hemisphere | | | | |
|  | Stress | Stress | Stress | Δstress T1-T2 | Δstress T2-T3 | Stress | Stress | Stress | Δstress T1-T2 | Δstress T2-T3 |
| Brain metrics | T1 | T2 | T3 | ΔT1-T2 | ΔT2-T3 | T1 | T2 | T3 | ΔT1-T2 | ΔT2-T3 |
| **Volume** |  |  |  |  |  |  |  |  |  |  |
| Middle frontal | -0.03 | -0.04 | -0.12 | -0.04 | -0.15*** | -0.02 | -0.01 | -0.07 | -0.02 | -0.01 |
| Orbital frontal | -0.04 | -0.06 | -0.06 | -0.04 | -0.11* | -0.02 | -0.06 | -0.08 | -0.07 | -0.06 |
| Anterior cingulate | -0.07 | -0.15*** | -0.11* | -0.06 | -0.13** | -0.04 | -0.01 | -0.12** | -0.01 | -0.03 |
| Inferior frontal | -0.02 | 0.02 | -0.01 | -0.05 | -0.04 | -0.05 | 0.00 | -0.07 | -0.04 | -0.04 |
| Frontal pole | -0.02 | 0.02 | -0.01 | -0.05 | -0.01 | -0.03 | 0.04 | -0.07 | -0.03 | -0.05 |
| Superior parietal | -0.08 | -0.03 | -0.07 | -0.05 | -0.01 | -0.06 | 0.00 | -0.06 | -0.04 | -0.02 |
| Superior frontal | -0.07 | 0.01 | -0.05 | -0.02 | -0.03 | -0.08 | -0.01 | -0.07 | -0.05 | -0.03 |
| Inferior parietal | -0.09 | -0.08 | -0.12** | -0.06 | -0.06 | -0.12** | -0.10* | -0.09 | -0.05 | -0.12** |
| Precuneus | -0.08 | -0.07 | -0.06 | -0.05 | 0.00 | -0.07 | -0.04 | -0.03 | -0.03 | 0.00 |
| Posterior cingulate | -0.06 | -0.08 | 0.00 | -0.06 | -0.04 | -0.09* | -0.03 | -0.04 | -0.02 | -0.05 |
| Insula | -0.05 | -0.04 | -0.04 | -0.05 | 0.00 | -0.07 | -0.06 | -0.03 | 0.02 | 0.03 |
| **Surface area** |  |  |  |  |  |  |  |  |  |  |
| Middle frontal | -0.02 | -0.09* | -0.09* | -0.03 | -0.08 | -0.02 | -0.08 | -0.05 | -0.03 | 0.01 |
| Orbital frontal | -0.02 | -0.07 | -0.07 | -0.01 | -0.10* | -0.02 | -0.10* | -0.05 | -0.06 | -0.09 |
| Anterior cingulate | -0.04 | -0.15** | -0.10* | -0.06 | -0.06 | -0.05 | -0.04 | -0.10* | -0.01 | -0.02 |
| Inferior frontal | -0.02 | -0.02 | -0.01 | -0.04 | 0.04 | -0.02 | -0.04 | -0.06 | -0.03 | 0.02 |
| Frontal pole | -0.04 | -0.08 | 0.03 | -0.04 | 0.04 | -0.04 | -0.05 | -0.03 | -0.02 | 0.01 |
| Superior parietal | -0.06 | -0.06 | -0.06 | -0.04 | 0.00 | -0.04 | -0.06 | -0.07 | -0.04 | 0.00 |
| Superior frontal | -0.05 | -0.05 | -0.02 | -0.02 | 0.04 | -0.07 | -0.09* | -0.06 | -0.06 | -0.03 |
| Inferior parietal | -0.06 | -0.10* | -0.11* | -0.04 | -0.01 | -0.12** | -0.10* | -0.08 | -0.03 | -0.04 |
| Precuneus | -0.07 | -0.06 | -0.07 | -0.06 | 0.01 | -0.07 | -0.08 | -0.04 | -0.03 | 0.01 |
| Posterior cingulate | -0.03 | -0.10 | -0.03 | -0.06 | 0.00 | -0.10* | -0.08 | -0.05 | -0.04 | 0.02 |
| Insula | -0.02 | -0.05 | -0.04 | -0.02 | -0.06 | -0.03 | -0.06 | -0.04 | -0.05 | 0.02 |
| **Cortical thickness** |  |  |  |  |  |  |  |  |  |  |
| Middle frontal | -0.04 | 0.08 | -0.07 | -0.03 | -0.08 | -0.02 | 0.10* | 0.00 | 0.00 | 0.00 |
| Orbital frontal | -0.10* | 0.00 | -0.01 | -0.03 | -0.06 | -0.01 | 0.10* | -0.04 | -0.02 | 0.02 |
| Anterior cingulate | -0.04 | -0.04 | 0.05 | 0.01 | -0.10* | -0.03 | 0.05 | -0.04 | -0.04 | -0.03 |
| Inferior frontal | -0.04 | 0.08 | -0.04 | -0.03 | -0.07 | -0.09 | 0.09* | -0.02 | -0.03 | -0.01 |
| Frontal pole | 0.03 | 0.10 | -0.05 | -0.02 | -0.06 | -0.01 | 0.10* | -0.08 | -0.06 | -0.03 |
| Superior parietal | -0.05 | 0.05 | 0.01 | -0.04 | 0.02 | -0.04 | 0.09* | 0.05 | -0.01 | 0.04 |
| Superior frontal | -0.07 | 0.09 | -0.05 | -0.02 | -0.06 | -0.01 | 0.14** | -0.04 | -0.03 | 0.00 |
| Inferior parietal | -0.05 | 0.06 | 0.02 | -0.02 | 0.00 | -0.04 | -0.05 | 0.05 | -0.03 | -0.05 |
| Precuneus | -0.07 | -0.03 | 0.00 | 0.02 | 0.02 | -0.06 | 0.08 | 0.04 | -0.01 | -0.01 |
| Posterior cingulate | -0.05 | 0.05 | 0.05 | 0.00 | -0.09* | 0.00 | 0.13** | 0.01 | -0.01 | -0.10* |
| Insula | -0.10* | -0.02 | 0.00 | -0.07 | 0.03 | -0.10* | 0.00 | -0.03 | -0.03 | -0.07 |
| **Cognition** |  |  |  |  |  |  |  |  |  |  |
| SWM Strategy | 0.02 | -0.06 | -0.02 | 0.04 | 0.01 |  |  |  |  |  |
| SWM Errors | -0.01 | -0.06 | -0.03 | -0.01 | -0.05 |  |  |  |  |  |
| CGT decision making | 0.02 | 0.06 | -0.06 | 0.07 | 0.04 |  |  |  |  |  |
| CGT risk taking | -0.02 | -0.06 | 0.12** | -0.04 | 0.00 |  |  |  |  |  |
| CGT risk adjustment | 0.00 | 0.00 | -0.13** | 0.07 | -0.01 |  |  |  |  |  |
| Note: *** = significant at p<0.001; ** = significant at p<0.01; * = significant at p<0.05 | | | | | | | | | | |

| Table S5. Complete longitudinal mediation analyses showing indirect effects of different brain regions (path 1) or cognitive functioning (path 2) in the relations between adolescent stress exposure, brain development and cognitive functioning. Bias corrected 95% confidence intervals indicate no significant mediating effect in any of the models or paths examined. | | | | | | | |
| --- | --- | --- | --- | --- | --- | --- | --- |
|  | Indirect effects | Brain Volume | | Surface area | | Cortical Thickness | |
|  |  | β | 95%CI | β | 95%CI | β | 95%CI |
|  | **SWM strategy** |  |  |  |  |  |  |
| 1st path | Stress T1 =>Middle frontal T2 => SWM strategy T3 | 0.003 | -0.001, 0.011 | 0.000 | -0.004, 0.005 | 0.000 | -0.010, 0.007 |
| 2^nd^ path | Stress T1 => SWM strategy T2 => Middle frontal T3 | 0.001 | -0.001, 0.005 | 0.000 | -0.001, 0.003 | 0.003 | -0.002, 0.012 |
| 1st path | Stress T1 =>Orbitofrontal T2 => SWM strategy T3 | 0.002 | -0.001, 0.012 | 0.000 | -0.005, 0.004 | 0.002 | -0.005, 0.012 |
| 2^nd^ path | Stress T1 => SWM strategy T2 => Orbitofrontal T3 | -0.001 | -0.005, 0.001 | -0.001 | -0.005, 0.001 | 0.000 | -0.003, 0.004 |
| 1st path | Stress T1 =>anterior cingulate T2 => SWM strategy T3 | 0.001 | -0.005, 0.009 | 0.000 | -0.006, 0.005 | 0.000 | -0.004, 0.006 |
| 2^nd^ path | Stress T1 => SWM strategy T2 =>anterior cingulate T3 | 0.001 | -0.001, 0.005 | 0.000 | -0.003, 0.001 | 0.002 | -0.001, 0.010 |
| 1st path | Stress T1 =>inferior frontal T2 => SWM strategy T3 | 0.001 | -0.002, 0.008 | 0.000 | -0.001, 0.004 | -0.001 | -0.011, 0.006 |
| 2^nd^ path | Stress T1 => SWM strategy T2 => inferior frontal T3 | 0.001 | -0.001, 0.005 | 0.000 | -0.002, 0.002 | 0.002 | -0.001, 0.012 |
| 1st path | Stress T1 =>frontal pole T2 => SWM strategy T3 | 0.000 | -0.003, 0.007 | -0.001 | -0.010, 0.001 | -0.001 | -0.010, 0.005 |
| 2^nd^ path | Stress T1 => SWM strategy T2 => frontal pole T3 | 0.001 | -0.001, 0.008 | -0.001 | -0.007, 0.001 | 0.002 | -0.001, 0.010 |
| 1st path | Stress T1 =>superior parietal T2 => SWM strategy T3 | -0.001 | -0.008, 0.005 | 0.000 | -0.004, 0.002 | 0.000 | -0.008, 0.007 |
| 2^nd^ path | Stress T1 => SWM strategy T2 => superior parietal T3 | 0.000 | -0.001, 0.005 | 0.000 | -0.001, 0.004 | 0.002 | -0.001, 0.009 |
| 1st path | Stress T1 =>superior frontal T2 => SWM strategy T3 | 0.001 | -0.002, 0.008 | 0.000 | -0.002, 0.004 | 0.001 | -0.008, 0.011 |
| 2^nd^ path | Stress T1 => SWM strategy T2 => superior frontalT3 | 0.001 | -0.001, 0.005 | -0.001 | -0.004, 0.000 | 0.003 | -0.002, 0.012 |
| 1st path | Stress T1 =>inferior parietal T2 => SWM strategy T3 | 0.000 | -0.001, 0.004 | 0.000 | -0.004, 0.001 | -0.001 | -0.010, 0.001 |
| 2^nd^ path | Stress T1 => SWM strategy T2 => inferior parietal T3 | 0.001 | -0.001, 0.005 | 0.000 | -0.001, 0.003 | 0.002 | -0.001, 0.011 |
| 1st path | Stress T1 =>Precuneus T2 => SWM strategy T3 | 0.000 | -0.003, 0.005 | 0.000 | -0.002, 0.004 | 0.000 | -0.002, 0.005 |
| 2^nd^ path | Stress T1 => SWM strategy T2 => Precuneus T3 | 0.000 | -0.001, 0.004 | 0.000 | -0.002, 0.001 | 0.001 | -0.001, 0.008 |
| 1st path | Stress T1 =>Posterior cingulate T2=>SWM strategy T3 | 0.000 | -0.001, 0.006 | 0.000 | -0.001, 0.004 | 0.002 | -0.001, 0.008 |
| 2^nd^ path | Stress T1 => SWM strategy T2=>posterior cingulate T3 | 0.001 | -0.001, 0.006 | 0.000 | -0.003, 0.002 | 0.001 | -0.001, 0.009 |
| 1st path | Stress T1 =>insula T2 => SWM strategy T3 | 0.000 | -0.002, 0.003 | 0.000 | -0.002, 0.002 | 0.001 | -0.001, 0.008 |
| 2^nd^ path | Stress T1 => SWM strategy T2 => insula T3 | 0.001 | -0.001, 0.005 | 0.000 | -0.001, 0.004 | 0.002 | -0.001, 0.010 |
|  |  |  |  |  |  |  |  |
|  | **SWM between errors** |  |  |  |  |  |  |
| 1st path | Stress T1 =>Middle frontal T2 => SWM errors T3 | 0.000 | -0.003, 0.007 | 0.000 | -0.002, 0.003 | 0.000 | -0.009, 0.008 |
| 2^nd^ path | Stress T1 => SWM errors T2 => Middle frontal T3 | 0.000 | -0.001, 0.002 | 0.000 | -0.001, 0.002 | 0.001 | -0.004, 0.007 |
| 1st path | Stress T1 =>Orbitofrontal T2 => SWM errors T3 | -0.001 | -0.010, 0.001 | 0.000 | -0.003, 0.004 | 0.000 | -0.008, 0.008 |
| 2^nd^ path | Stress T1 => SWM errors T2 => Orbitofrontal T3 | 0.000 | -0.003, 0.001 | 0.000 | -0.001, 0.002 | 0.000 | -0.005, 0.002 |
| 1st path | Stress T1 =>anterior cingulate T2 => SWM errors T3 | 0.000 | -0.002, 0.007 | 0.000 | -0.003, 0.002 | 0.002 | -0.001, 0.009 |
| 2^nd^ path | Stress T1 => SWM errors T2 =>anterior cingulate T3 | 0.000 | -0.002, 0.002 | 0.000 | -0.002, 0.001 | 0.000 | -0.002, 0.005 |
| 1st path | Stress T1 =>inferior frontal T2 => SWM errors T3 | -0.001 | -0.007, 0.002 | 0.000 | -0.003, 0.002 | -0.002 | -0.012, 0.004 |
| 2^nd^ path | Stress T1 => SWM errors T2 => inferior frontal T3 | 0.000 | -0.002, 0.001 | 0.000 | -0.002, 0.001 | 0.000 | -0.002, 0.007 |
| 1st path | Stress T1 =>frontal pole T2 => SWM errors T3 | 0.000 | -0.008, 0.003 | 0.000 | -0.007, 0.002 | -0.002 | -0.013, 0.005 |
| 2^nd^ path | Stress T1 => SWM errors T2 => frontal pole T3 | 0.000 | -0.002, 0.005 | 0.000 | -0.003, 0.002 | 0.000 | -0.002, 0.004 |
| 1st path | Stress T1 =>superior parietal T2 => SWM errors T3 | -0.001 | -0.010, 0.005 | 0.000 | -0.005, 0.002 | -0.001 | -0.009, 0.006 |
| 2^nd^ path | Stress T1 => SWM errors T2 => superior parietal T3 | 0.000 | -0.004, 0.001 | 0.000 | -0.004, 0.001 | 0.000 | -0.002, 0.005 |
| 1st path | Stress T1 =>superior frontal T2 => SWM errors T3 | -0.003 | -0.013, 0.001 | 0.000 | -0.005, 0.002 | -0.002 | -0.012, 0.007 |
| 2^nd^ path | Stress T1 => SWM errors T2 => superior frontal T3 | 0.000 | -0.002, 0.001 | 0.000 | -0.003, 0.001 | 0.001 | -0.003, 0.008 |
| 1st path | Stress T1 =>inferior parietal T2 => SWM errors T3 | 0.000 | -0.005, 0.001 | 0.000 | -0.001, 0.003 | -0.002 | -0.012, 0.001 |
| 2^nd^ path | Stress T1 => SWM errors T2 => inferior parietal T3 | 0.000 | -0.001, 0.002 | 0.000 | -0.001, 0.002 | 0.000 | -0.002, 0.005 |
| 1st path | Stress T1 =>Precuneus T2 => SWM errors T3 | -0.001 | -0.008, 0.002 | -0.001 | -0.007, 0.001 | 0.000 | -0.001, 0.006 |
| 2^nd^ path | Stress T1 => SWM errors T2 => Precuneus T3 | 0.000 | -0.001, 0.003 | 0.000 | -0.001, 0.002 | 0.000 | -0.003, 0.005 |
| 1st path | Stress T1 =>Posterior cingulate T2=>SWM errors T3 | 0.000 | -0.001, 0.004 | 0.000 | -0.003, 0.002 | 0.002 | -0.001, 0.012 |
| 2^nd^ path | Stress T1 => SWM errors T2=>posterior cingulate T3 | 0.000 | -0.002, 0.004 | 0.000 | -0.002, 0.001 | 0.001 | -0.007, 0.009 |
| 1st path | Stress T1 =>insula T2 => SWM errors T3 | 0.000 | -0.004, 0.001 | 0.000 | -0.001, 0.004 | 0.000 | -0.007, 0.002 |
| 2^nd^ path | Stress T1 => SWM errors T2 => insula T3 | 0.000 | -0.002, 0.001 | 0.000 | -0.003, 0.001 | 0.000 | -0.003, 0.005 |
|  |  |  |  |  |  |  |  |
|  | **CGT quality of decision making (QDM)** |  |  |  |  |  |  |
| 1st path | Stress T1 =>Middle frontal T2 => CGT QDM T3 | 0.001 | -0.002, 0.009 | 0.000 | -0.003, 0.002 | 0.003 | -0.004, 0.013 |
| 2^nd^ path | Stress T1 => CGT QDM T2 => Middle frontal T3 | -0.001 | -0.006, 0.001 | 0.000 | -0.003, 0.001 | 0.000 | -0.008, 0.003 |
| 1st path | Stress T1 =>Orbitofrontal T2 => CGT QDM T3 | 0.001 | -0.001, 0.008 | 0.000 | -0.002, 0.002 | 0.004 | -0.001, 0.014 |
| 2^nd^ path | Stress T1 => CGT QDM T2 => Orbitofrontal T3 | -0.002 | -0.008, 0.001 | 0.000 | -0.007, 0.001 | -0.001 | -0.009, 0.001 |
| 1st path | Stress T1 =>anterior cingulate T2 => CGT QDM T3 | 0.001 | -0.002, 0.006 | 0.000 | -0.003, 0.002 | 0.002 | -0.001, 0.010 |
| 2^nd^ path | Stress T1 => CGT QDM T2 =>anterior cingulate T3 | -0.001 | -0.005, 0.001 | -0.001 | -0.004, 0.001 | 0.000 | -0.003, 0.008 |
| 1st path | Stress T1 =>inferior frontal T2 => CGT QDM T3 | 0.000 | -0.003, 0.006 | 0.000 | -0.003, 0.001 | 0.002 | -0.003, 0.013 |
| 2^nd^ path | Stress T1 => CGT QDM T2 => inferior frontal T3 | -0.002 | -0.007, 0.001 | -0.001 | -0.005, 0.001 | -0.001 | -0.010, 0.001 |
| 1st path | Stress T1 =>frontal pole T2 => CGT QDM T3 | 0.001 | -0.001, 0.008 | 0.000 | -0.004, 0.002 | 0.000 | -0.007, 0.008 |
| 2^nd^ path | Stress T1 => CGT QDM T2 => frontal pole T3 | -0.002 | -0.010, 0.001 | -0.002 | -0.010, 0.001 | 0.000 | -0.007, 0.002 |
| 1st path | Stress T1 =>superior parietal T2 => CGT QDM T3 | 0.000 | -0.006, 0.006 | -0.001 | -0.005, 0.001 | 0.003 | -0.002, 0.014 |
| 2^nd^ path | Stress T1 => CGT QDM T2 => superior parietal T3 | -0.001 | -0.007, 0.002 | -0.001 | -0.005, 0.001 | -0.001 | -0.008, 0.002 |
| 1st path | Stress T1 =>superior frontal T2 => CGT QDM T3 | -0.001 | -0.007, 0.002 | 0.000 | -0.003, 0.001 | -0.001 | -0.011, 0.007 |
| 2^nd^ path | Stress T1 => CGT QDM T2 => superior frontal T3 | -0.001 | -0.008, 0.001 | -0.001 | -0.004, 0.001 | -0.001 | -0.011, 0.001 |
| 1st path | Stress T1 =>inferior parietal T2 => CGT QDM T3 | 0.000 | -0.003, 0.002 | 0.000 | -0.001, 0.004 | 0.002 | -0.001, 0.012 |
| 2^nd^ path | Stress T1 => CGT QDM T2 => inferior parietal T3 | -0.001 | -0.007, 0.002 | -0.001 | -0.004, 0.001 | -0.001 | -0.011, 0.001 |
| 1st path | Stress T1 =>Precuneus T2 => CGT QDM T3 | 0.000 | -0.005, 0.002 | -0.001 | -0.005, 0.001 | 0.001 | -0.002, 0.008 |
| 2^nd^ path | Stress T1 => CGT QDM T2 => Precuneus T3 | -0.001 | -0.006, 0.001 | -0.001 | -0.004, 0.001 | -0.001 | -0.009, 0.001 |
| 1st path | Stress T1 =>Posterior cingulate T2=> CGT QDM T3 | 0.000 | -0.005, 0.001 | 0.000 | -0.004, 0.001 | 0.000 | -0.002, 0.006 |
| 2^nd^ path | Stress T1 => CGT QDM T2=>posterior cingulate T3 | -0.002 | -0.008, 0.001 | -0.001 | -0.007, 0.001 | -0.001 | -0.009, 0.002 |
| 1st path | Stress T1 =>insula T2 => CGT QDM T3 | 0.000 | -0.001, 0.003 | 0.000 | -0.001, 0.003 | 0.001 | -0.001, 0.007 |
| 2^nd^ path | Stress T1 => CGT QDM T2 => insula T3 | -0.001 | -0.006, 0.001 | -0.001 | -0.006, 0.001 | -0.001 | -0.008, 0.001 |
|  |  |  |  |  |  |  |  |
|  | **CGT risk taking** |  |  |  |  |  |  |
| 1st path | Stress T1 =>Middle frontal T2 => CGT QDM T3 | 0.000 | -0.005, 0.004 | 0.000 | -0.002, 0.004 | -0.004 | -0.015, 0.002 |
| 2^nd^ path | Stress T1 => CGT risk taking T2 => Middle frontal T3 | -0.001 | -0.007, 0.001 | -0.002 | -0.007, 0.002 | 0.002 | -0.001, 0.011 |
| 1st path | Stress T1 =>Orbitofrontal T2 => CGT risk taking T3 | -0.001 | -0.008, 0.002 | 0.000 | -0.003, 0.002 | -0.004 | -0.015, 0.001 |
| 2^nd^ path | Stress T1 => CGT risk taking T2 => Orbitofrontal T3 | 0.000 | -0.004, 0.001 | 0.000 | -0.004, 0.001 | 0.000 | -0.002, 0.007 |
| 1st path | Stress T1=>anterior cingulate T2 =>CGT risk taking T3 | 0.000 | -0.001, 0.005 | 0.000 | -0.003, 0.002 | 0.000 | -0.007, 0.003 |
| 2^nd^ path | Stress T1=>CGT risk taking T2 =>anterior cingulate T3 | -0.001 | -0.005, 0.001 | -0.001 | -0.005, 0.001 | 0.000 | -0.003, 0.006 |
| 1st path | Stress T1 =>inferior frontal T2 => CGT risk taking T3 | -0.002 | -0.009, 0.001 | 0.000 | -0.005, 0.001 | -0.002 | -0.012, 0.004 |
| 2^nd^ path | Stress T1 => CGT risk taking T2 => inferior frontal T3 | -0.001 | -0.005, 0.001 | -0.001 | -0.005, 0.001 | 0.001 | -0.001, 0.007 |
| 1st path | Stress T1 =>frontal pole T2 => CGT risk taking T3 | 0.001 | -0.001, 0.010 | -0.001 | -0.007, 0.001 | -0.001 | -0.008, 0.006 |
| 2^nd^ path | Stress T1 => CGT risk taking T2 => frontal pole T3 | 0.000 | -0.003, 0.005 | -0.001 | -0.009, 0.001 | 0.001 | -0.001, 0.007 |
| 1st path | Stress T1 =>superior parietal T2 => CGT risk taking T3 | 0.002 | -0.004, 0.010 | 0.001 | -0.001, 0.007 | -0.001 | -0.010, 0.005 |
| 2^nd^ path | Stress T1=> CGT risk taking T2 => superior parietal T3 | 0.000 | -0.002, 0.005 | 0.000 | -0.002, 0.003 | 0.000 | -0.003, 0.005 |
| 1st path | Stress T1 =>superior frontal T2 => CGT risk taking T3 | -0.001 | -0.006, 0.003 | 0.000 | -0.002, 0.004 | -0.007 | -0.020, -0.001 |
| 2^nd^ path | Stress T1 => CGT risk taking T2 => superior frontal T3 | 0.000 | -0.004, 0.001 | -0.001 | -0.004, 0.001 | 0.001 | -0.001, 0.009 |
| 1st path | Stress T1 =>inferior parietal T2 => CGT risk taking T3 | 0.000 | -0.001, 0.005 | 0.000 | -0.004, 0.001 | -0.002 | -0.012, 0.001 |
| 2^nd^ path | Stress T1 => CGT risk taking T2 => inferior parietal T3 | 0.000 | -0.004, 0.001 | 0.000 | -0.004, 0.000 | 0.000 | -0.006, 0.002 |
| 1st path | Stress T1 =>Precuneus T2 => CGT risk taking T3 | 0.000 | -0.005, 0.003 | 0.000 | -0.004, 0.002 | 0.000 | -0.004, 0.002 |
| 2^nd^ path | Stress T1 => CGT risk taking T2 => Precuneus T3 | 0.000 | -0.003, 0.001 | 0.000 | -0.001, 0.003 | -0.001 | -0.008, 0.001 |
| 1st path | Stress T1=>Posterior cingulate T2=>CGT risk taking T3 | 0.001 | -0.001, 0.007 | 0.001 | -0.002, 0.006 | 0.000 | -0.002, 0.005 |
| 2^nd^ path | Stress T1=>CGT risk taking T2=>posterior cingulate T3 | 0.000 | -0.004, 0.001 | -0.001 | -0.006, 0.001 | 0.001 | -0.001, 0.010 |
| 1st path | Stress T1 =>insula T2 => CGT risk taking T3 | 0.000 | -0.002, 0.002 | 0.000 | -0.004, 0.001 | -0.001 | -0.007, 0.002 |
| 2^nd^ path | Stress T1 => CGT risk taking T2 => insula T3 | 0.000 | -0.001, 0.005 | 0.000 | -0.004, 0.002 | 0.002 | -0.001, 0.011 |
|  |  |  |  |  |  |  |  |
|  | **CGT risk adjustment** |  |  |  |  |  |  |
| 1st path | Stress T1 =>Middle frontal T2 => CGT risk adjust T3 | 0.000 | -0.007, 0.003 | 0.000 | -0.004, 0.003 | 0.003 | -0.003, 0.013 |
| 2^nd^ path | Stress T1 => CGT risk adjust T2 => Middle frontal T3 | 0.000 | -0.003, 0.004 | 0.000 | -0.004, 0.002 | 0.001 | -0.001, 0.009 |
| 1st path | Stress T1 =>Orbitofrontal T2 => CGT risk adjust T3 | 0.000 | -0.002, 0.006 | 0.000 | -0.002, 0.003 | 0.004 | -0.001, 0.013 |
| 2^nd^ path | Stress T1 => CGT risk adjust T2 => Orbitofrontal T3 | -0.001 | -0.006, 0.001 | -0.001 | -0.006, 0.001 | 0.002 | -0.001, 0.009 |
| 1st path | Stress T1=>anterior cingulate T2 =>CGT risk adjust T3 | 0.000 | -0.004, 0.001 | 0.000 | -0.002, 0.003 | 0.003 | -0.001, 0.011 |
| 2^nd^ path | Stress T1=>CGT risk adjust T2 =>anterior cingulate T3 | -0.001 | -0.008, 0.001 | -0.002 | -0.008, 0.000 | 0.004 | -0.001, 0.014 |
| 1st path | Stress T1 =>inferior frontal T2 => CGT risk adjust T3 | 0.000 | -0.004, 0.005 | 0.000 | -0.003, 0.001 | 0.002 | -0.005, 0.010 |
| 2^nd^ path | Stress T1 => CGT risk adjust T2 => inferior frontal T3 | -0.001 | -0.007, 0.001 | -0.002 | -0.008, 0.000 | 0.002 | -0.001, 0.010 |
| 1st path | Stress T1 =>frontal pole T2 => CGT risk adjust T3 | 0.001 | -0.001, 0.009 | -0.001 | -0.007, 0.001 | 0.001 | -0.005, 0.008 |
| 2^nd^ path | Stress T1 => CGT risk adjust T2 => frontal pole T3 | -0.001 | -0.010, 0.001 | -0.004 | -0.014, 0.001 | 0.001 | -0.001, 0.008 |
| 1st path | Stress T1 =>superior parietal T2 => CGT risk adjust T3 | 0.000 | -0.006, 0.006 | 0.000 | -0.005, 0.001 | 0.002 | -0.004, 0.009 |
| 2^nd^ path | Stress T1=> CGT risk adjust T2 => superior parietal T3 | -0.002 | -0.008, 0.001 | -0.002 | -0.006, 0.000 | -0.001 | -0.007, 0.001 |
| 1st path | Stress T1 =>superior frontal T2 => CGT risk adjust T3 | 0.000 | -0.004, 0.006 | 0.000 | -0.003, 0.002 | 0.002 | -0.005, 0.012 |
| 2^nd^ path | Stress T1 => CGT risk adjust T2 => superior frontal T3 | -0.001 | -0.007, 0.001 | -0.001 | -0.005, 0.000 | 0.001 | -0.002, 0.008 |
| 1st path | Stress T1 =>inferior parietal T2 => CGT risk adjust T3 | 0.000 | -0.001, 0.005 | 0.000 | -0.003, 0.001 | 0.001 | -0.002, 0.008 |
| 2^nd^ path | Stress T1 => CGT risk adjust T2 => inferior parietal T3 | -0.002 | -0.008, 0.001 | -0.001 | -0.005, 0.001 | -0.002 | -0.011, 0.001 |
| 1st path | Stress T1 =>Precuneus T2 => CGT risk adjust T3 | 0.000 | -0.002, 0.005 | 0.000 | -0.004, 0.001 | 0.000 | -0.002, 0.006 |
| 2^nd^ path | Stress T1 => CGT risk adjust T2 => Precuneus T3 | -0.001 | -0.006, 0.001 | -0.001 | -0.006, 0.000 | 0.000 | -0.004, 0.003 |
| 1st path | Stress T1=>Posterior cingulate T2=>CGT risk adjust T3 | -0.001 | -0.007, 0.002 | -0.001 | -0.006, 0.003 | 0.000 | -0.002, 0.005 |
| 2^nd^ path | Stress T1=>CGT risk adjust T2=>posterior cingulate T3 | -0.002 | -0.010, 0.001 | -0.003 | -0.010, 0.001 | 0.002 | -0.001, 0.010 |
| 1st path | Stress T1 =>insula T2 => CGT risk adjust T3 | 0.000 | -0.002, 0.002 | 0.000 | -0.001, 0.005 | 0.001 | -0.001, 0.009 |
| 2^nd^ path | Stress T1 => CGT risk adjust T2 => insula T3 | -0.002 | -0.008, 0.001 | -0.002 | -0.008, 0.001 | 0.001 | -0.002, 0.007 |
| Cl = Confidence intervals; CGT = Cambridge Gambling Task; QDM = Quality of Decision Making; risk adjust = risk adjustment; T1 = Timepoint 1; Time 2= Timepoint 2; T3 = Timepoint 3 | | | | | | | |

***Sensitivity analyses***

We reanalyzed the bivariate latent change models that examined the longitudinal association between stress and cortical structures by including the total Gray Matter volume as a covariate in the model. Results of the sensitivity analyses showed that the associations did not substantially vary from the results of the primary analyses that did not include the total Gray Matter volume as covariate. With the exception of left superior frontal volume which showed significant reduction across ages 14-22 as a result of baseline stress at age 14, no other inconsistency was observed between the sensitivity analyses and the primary analyses. As was the case with primary analyses, no result in the sensitivity analyses survived correction for multiple comparisons. Results of the sensitivity analyses are shown in Table S6 for model examining stress and brain volume, Table S7 for model examining cortical surface area and Table S8 for model examining cortical thickness.

| Table S6: Bivariate latent change score model examining the relations between adolescent stress exposure and brain volume, controlling for Total Gray Matter volume | | | | |
| --- | --- | --- | --- | --- |
|  | Left Hemisphere | | Right Hemisphere | |
|  | Lagged effects of stress  γ1 | Lagged effects of brain volume  γ2 | Lagged effects of stress  γ1 | Lagged effects of brain volume  γ2 |
|  | Std. *β* (SE) | Std. β (SE) | Std. *β* (SE) | Std. *β* (SE) |
| Middle frontal | -0.717 (0.526) | 0.051 (0.228) | -0.208 (0.188) | 0.072 (0.168) |
| Orbital frontal | -0.290 (0.343) | 0.076 (0.200) | 0.023 (0.245) | 0.049 (0.167) |
| Anterior cingulate | -0.277 (0.363) | 0.084 (0.363) | -0.348 (0.164)* | 0.098 (0.200) |
| Inferior frontal | -0.231 (0.236) | 0.178 (0.250) | -0.266 (0.226) | 0.154 (0.231) |
| Frontal pole | 0.319 (0.488) | -0.045 (0.439) | -0.296 (0.382) | -0.225 (0.392) |
| Superior parietal | -0.227 (0.633) | 0.279 (0.586) | -0.289 (0.283) | 0.259 (0.343) |
| Superior frontal | -0.449 (0.246) | 0.013 (0.205) | -0.196 (0.251) | 0.082 (0.183) |
| Inferior parietal | -0.571 (0.433) | 0.294 (0.428) | -0.156 (0.266) | -0.040 (0.287) |
| Precuneus | 0.179 (0.414) | 0.263 (0.367) | 0.211 (0.348) | -0.029 (0.333) |
| Posterior cingulate | -0.036 (0.170) | 0.502 (0.396) | -0.309 (0.280) | 0.127 (0.359) |
| Insula | 0.171 (0.186) | 0.188 (0.323) | 0.051 (0.215) | -0.222 (0.346) |
| Note: * = significant at p<0.05; SE = Standard Error. | | | | |

| Table S7: Bivariate latent change score model examining the relations between adolescent stress exposure and brain surface area, controlling for Total Gray Matter volume | | | | |
| --- | --- | --- | --- | --- |
|  | Left Hemisphere | | Right Hemisphere | |
|  | Lagged effects of stress  γ1 | Lagged effects of brain surface area  γ2 | Lagged effects of stress  γ1 | Lagged effects of brain surface area  γ2 |
|  | Std. *β* (SE) | Std. β (SE) | Std. *β* (SE) | Std. *β* (SE) |
| Middle frontal | -0.382 (0.230) | 0.178 (0.158) | -0.194 (0.151) | 0.278 (0.189) |
| Orbital frontal | -0.545 (0.325) | 0.144 (0.177) | -0.068 (0.042) | 0.030 (0.144) |
| Anterior cingulate | -0.101 (0.192) | 0.193 (0.361) | -0.290 (0.131)* | 0.360 (0.267) |
| Inferior frontal | 0.073 (0.145) | 0.207 (0.192) | -0.121 (0.182) | 0.309 (0.260) |
| Frontal pole | 0.691 (0.576) | 0.428 (0.792) | 0.152 (0.245) | 0.098 (0.386) |
| Superior parietal | -0.115 (0.589) | 0.145 (0.341) | -0.048 (0.297) | 0.177 (0.553) |
| Superior frontal | -0.251 (0.122)* | 0.251 (0.182) | -0.130 (0.158) | 0.332 (0.266) |
| Inferior parietal | -0.179 (0.260) | 0.254 (0.330) | -0.004 (0.195) | -0.069 (0.355) |
| Precuneus | 0.351 (0.724) | 0.232 (0.432) | -0.115 (0.704) | 0.057 (0.355) |
| Posterior cingulate | -0.079 (0.175) | 0.458 (0.287) | 0.020 (0.230) | 0.692 (0.689) |
| Insula | 0.088 (0.351) | -0.285 (0.917) | 0.040 (0.422) | -0.367 (0.589) |
| Note: * = significant at p<0.05; SE = Standard Error. | | | | |

| Table S8: Bivariate latent change score model of adolescent stress and brain cortical thickness, controlling for Total Gray Matter volume | | | | |
| --- | --- | --- | --- | --- |
|  | Left Hemisphere | | Right Hemisphere | |
|  | Lagged effects of stress  γ1 | Lagged effects of cortical thickness  γ2 | Lagged effects of stress  γ1 | Lagged effects of cortical thickness  γ2 |
|  | Std. *β* (SE) | Std. β (SE) | Std. *β* (SE) | Std. *β* (SE) |
| Middle frontal | -0.494 (0.549) | -0.106 (0.157) | 0.108 (0.306) | -0.123 (0.184) |
| Orbital frontal | 0.356 (0.310) | -0.622 (0.4110 | 0.305 (0.853) | -0.313 (0.208) |
| Anterior cingulate | 0.065 (0.047) | -0.032 (0.077) | 0.050 (0.291) | -0.164 (0.175) |
| Inferior frontal | -0.440 (0.403) | 0.556 (0.924) | -0.089 (0.410) | -0.037 (0.289) |
| Frontal pole | -0.070 (0.323) | -0.451 (0.403) | -0.501 (0.481) | 0.108 (0.393) |
| Superior parietal | -0.110 (0.416) | 0.199 (0.262) | -0.092 (0.249) | 0.163 (0.192) |
| Superior frontal | -0.254 (0.336) | -0.131 (0.139) | -0.167 (0.326) | -0.055 (0.200) |
| Inferior parietal | -0.580 (0.712) | 0.182 (0.337) | -0.164 (0.412) | 0.210 (0.222) |
| Precuneus | -0.011 (0.344) | 0.095 (0.275) | 0.165 (0.207) | 0.006 (0.152) |
| Posterior cingulate | -0.254 (0.428) | -0.576 (0.540) | 0.040 (0.035) | -0.003 (0.066) |
| Insula | 0.003 (0.466) | 0.875 (0.749) | -0.637 (0.690) | 0.077 (0.429) |
| Note: SE = Standard Error | | | | |
